# Anal High-Grade Squamous Intraepithelial Lesions in People Living with HIV: A Systematic Review of Treatment Outcomes

**DOI:** 10.3390/cancers18162577

**Published:** 2026-08-11

**Authors:** Hendrik Dapper, Felix Aigner, Christoph Stephan, Claudia Rudroff, Daniel Martin, Franz Rödel, Bruce Minsky, Ethan B. Ludmir, Craig Messick, Caleah Kitchens, Paul B. Romesser, Julio Garcia-Aguilar, J. Joshua Smith, Claus Rödel, Ulrike Wieland, Luisa Bopp, Emmanouil Fokas

**Affiliations:** 1 Department of Radiation Oncology, Cyberknife and Radiation Therapy, Center for Integrated Oncology Aachen Bonn Cologne Duesseldorf (CIO ABCD), Faculty of Medicine and University Hospital of Cologne, University of Cologne, 50937 Cologne, Germany; 2 Department of Surgery, Barmherzige Brüder Hospital Graz, 8020 Graz, Austria; felix.aigner@bbgraz.at; 3 Medical Department II, Section Infectious Diseases, University Medical Center, 60596 Frankfurt, Germany; 4 Clinic of Urology, University Hospital Cologne, 50937 Cologne, Germany; claudia.rudroff2@uk-koeln.de; 5 Department of Radiation Oncology, University Hospital, Johann Wolfgang Goethe University, 60590 Frankfurt, Germanyf.roedel@med.uni-frankfurt.de (F.R.);; 6 Frankfurt Cancer Institute, 60596 Frankfurt, Germany; 7 German Cancer Research Center (DKFZ), 69120 Heidelberg, Germany; 8 German Cancer Consortium (DKTK), Partner Site Frankfurt a. M., University of Frankfurt, 69120 Heidelberg, Germany; 9 Department of Gastrointestinal Radiation Oncology, The University of Texas MD Anderson Cancer Center, Houston, TX 77030, USA; 10 Department of Colon and Rectal Surgery, The University of Texas MD Anderson Cancer Center, Houston, TX 77030, USA; 11 Department of Surgery, Colorectal Service, Memorial Sloan Kettering Cancer Center, 1275 York Ave., New York, NY 10065, USAgarciaaj@mskcc.org (J.G.-A.);; 12 Department of Radiation Oncology, Colorectal and Anal Cancer Service, Memorial Sloan Kettering Cancer Center, New York, NY 10065, USA; romessep@mskcc.org; 13 Institute of Virology, National Reference Center for Papilloma and Polyomaviruses, University of Cologne, Faculty of Medicine and University Hospital Cologne, 50935 Cologne, Germany; ulrike.wieland@uni-koeln.de; 14 Department of Dermatology and Venereology, University of Cologne, Faculty of Medicine and University Hospital of Cologne, 50937 Cologne, Germany

**Keywords:** anal cancer prevention, high-grade squamous intraepithelial lesions, human papillomavirus, HIV infection

## Abstract

**Background/Objectives:** People living with HIV (PLWH) are at substantially increased risk of anal high-grade squamous intraepithelial lesions (HSIL), a recognized precursor of anal cancer. Although the ANCHOR trial demonstrated that treatment reduces progression to invasive disease, important uncertainties remain regarding durability of response, recurrence patterns, and treatment-related toxicity across available therapeutic strategies. **Methods:** We conducted a systematic review of studies evaluating treatment outcomes for anal HSIL published between 2000 and 2026. PubMed, Embase, and the Cochrane Library were searched according to PRISMA guidelines, with a particular focus on studies involving PLWH. **Results**: A total of 45 studies comprising 6093 patients, including five randomized controlled trials, met the inclusion criteria. Definitions of treatment response, recurrence, and adverse events varied considerably across studies, together with differences in assessment methods and follow-up, limiting direct comparisons between treatment modalities. Ablative, topical, and surgical approaches achieved variable short-term clearance; however, no modality demonstrated consistent durable disease control. Recurrence rates were high, frequently exceeding 50% in PLWH, consistent with the chronic and relapsing nature of HPV-driven disease in this population. Serious adverse events were uncommon, although treatment-related morbidity was frequent and varied across modalities. **Conclusions**: Randomized evidence supports treatment of biopsy-confirmed anal HSIL in PLWH for prevention of squamous cell carcinoma of the anus. However, comparative evidence remains limited by few randomized trials, heterogeneous study designs, and inconsistent outcome reporting. High recurrence rates and limited durability of response support the need for improved therapeutic strategies, biomarker-driven risk stratification, and longitudinal management approaches.

## Simple Summary

People living with HIV have a substantially increased risk of developing precancerous lesions in the anal canal that can progress to anal cancer. Recent research has shown that treating these lesions can reduce the risk of cancer development. However, it remains unclear which treatment provides the most durable long-term benefit, and the available comparative evidence remains limited. In this systematic review, we analyzed 45 studies involving more than 6000 patients to evaluate the effectiveness, recurrence rates, and side effects of currently available treatments. We found that while several treatment approaches can successfully remove or control lesions in the short term, recurrence is common, and no treatment consistently achieves long-lasting disease control. Serious side effects were uncommon, but treatment-related symptoms occurred frequently. Overall, the available evidence is limited by relatively few randomized studies and substantial differences in study design and outcome reporting. Our findings suggest that these precancerous lesions should be managed as a chronic condition requiring long-term follow-up. Future research should focus on developing more effective treatments and improving identification of patients at greatest risk of progression to cancer.

## 1. Introduction

Anal high-grade squamous intraepithelial lesions (HSIL) represent a major oncogenic complication in people living with HIV (PLWH), driven by persistent high-risk human papillomavirus (HPV) infection, particularly HPV16, that may progress to squamous cell carcinoma of the anus (SCCA) if untreated [1,2,3,4]. Anal precursor lesions are classified according to the Lower Anogenital Squamous Terminology (LAST) two-tier LSIL/HSIL framework, which harmonizes histological and cytological assessment across the lower anogenital tract [5,6].

Although SCCA remains rare in the general population, with age-standardized incidence rates of approximately 1–2 per 100,000 person-years in the United States, its incidence has increased over recent decades [7]. Rates are substantially higher in defined high-risk populations, especially PLWH, reaching 30–100 per 100,000 person-years among men who have sex with men (MSM) living with HIV [7,8]. Population-based incidence data for HSIL are limited; disease burden is therefore primarily characterized in high-risk groups, where HSIL prevalence frequently exceeds 20% among MSM living with HIV [4,9,10]. Registry-based analyses, predominantly of AIN III, report progression rates of 7–9% within several years of diagnosis, with higher risk among PLWH [11,12]. Together, these data position HSIL as a central target for SCCA prevention.

In PLWH, screening strategies have gained increasing attention, whereas accumulating data over the past decade have allowed clearer definition of high-risk populations eligible for targeted screening [13]. Modelling analyses in MSM living with HIV aged ≥35 years estimate that 331 high-resolution anoscopies (HRA) and 153 HSIL treatments are required to prevent one SCCA with annual screening—numbers comparable to established colorectal cancer prevention strategies [2,14]. Accordingly, international guidelines recommend risk-adapted diagnostic pathways rather than population-wide screening [13,15]. Anal cytology is commonly used in high-risk populations, whereas HRA with targeted biopsy remains the diagnostic reference standard [16,17].

Anal cancer prevention strategies encompass primary, secondary, and tertiary approaches. However, vaccine effectiveness is reduced in PLWH, and most individuals present after HPV exposure. Prophylactic HPV vaccination reduces anal HPV infection and high-grade intraepithelial neoplasia when administered prior to HPV exposure [18,19]. In a randomized trial among young MSM, quadrivalent HPV vaccination reduced the incidence of vaccine-type anal HSIL by approximately 75% and persistent infection with HPV vaccine types by 95% in the per-protocol analyses [20]. Secondary prevention focuses on early detection of HSIL through risk-adapted screening strategies, most commonly using anal cytology followed by HRA with targeted biopsy as the diagnostic reference standard.

While HPV vaccination and risk-adapted screening aim to prevent or detect anal precancerous lesions early, treatment of established HSIL represents the key strategy to prevent progression to invasive SCCA. The Anal Cancer/HSIL Outcomes Research (ANCHOR) trial conducted exclusively in PLWH, demonstrated that treatment of biopsy-confirmed HSIL in PLWH reduced progression to invasive SCCA by 57% compared with active monitoring (hazard ratio 0.43, 95% CI 0.20–0.93) [2]. However, beyond ANCHOR, the comparative effectiveness, durability, recurrence dynamics, and methodological robustness of available treatment strategies remain uncertain.

Previous reviews summarized therapeutic approaches for anal HSIL; however, most predate the ANCHOR trial or did not systematically assess recurrence patterns, durability of response, comparative toxicity, and methodological quality across modalities [13,21]. The present review provides an updated synthesis of the parameters as mentioned above. Given the disproportionate burden of HSIL and SCCA in PLWH, and the central role of HIV-related immunosuppression in disease persistence and recurrence, we conducted a systematic review to evaluate the efficacy, durability, recurrence patterns, safety, and comparative effectiveness of therapeutic strategies for anal HSIL.

## 2. Methods

### 2.1. Search Strategy and Selection Criteria

We carried out a systematic review in accordance with PRISMA 2020 [22]. The PRISMA checklist can be found in Supplementary Table S1. The review protocol was not prospectively registered. We searched PubMed, Embase, and the Cochrane Library for studies published from 1 January 2000 to 31 January 2026. The full electronic search strategy, including Boolean terms and database-specific syntax, is provided in the Supplementary Material. We also screened reference lists of eligible studies and relevant clinical guidelines to identify additional records.

We included randomized controlled trials (RCTs) and non-randomized studies enrolling at least 20 participants with biopsy-confirmed HSIL that reported treatment outcomes, including response, recurrence, or progression to SCCA. RCTs including mixed-grade populations (AIN I–III) were eligible if HSIL-specific outcomes were reported or if HSIL constituted the majority of treated lesions. Case reports, conference abstracts, letters, and duplicate publications were excluded.

### 2.2. Study Selection and Data Extraction

Two reviewers independently screened titles, abstracts, and full texts according to predefined eligibility criteria, with discrepancies resolved by consensus. The data extracted included study design, population characteristics (including HIV status and CD4 count where available), interventions and comparators, endpoints, response and recurrence rates, adverse events, and duration of follow-up.

### 2.3. Risk of Bias Assessment

Risk of bias in RCTs was assessed using the Cochrane Risk of Bias 2 (RoB 2) tool, and prospective non-randomized studies were evaluated using the Risk Of Bias In Non-Randomized Studies of Interventions (ROBINS-I) tool. Assessments were performed independently by two reviewers, with discrepancies resolved by discussion.

### 2.4. Data Synthesis

**Figure 1:**
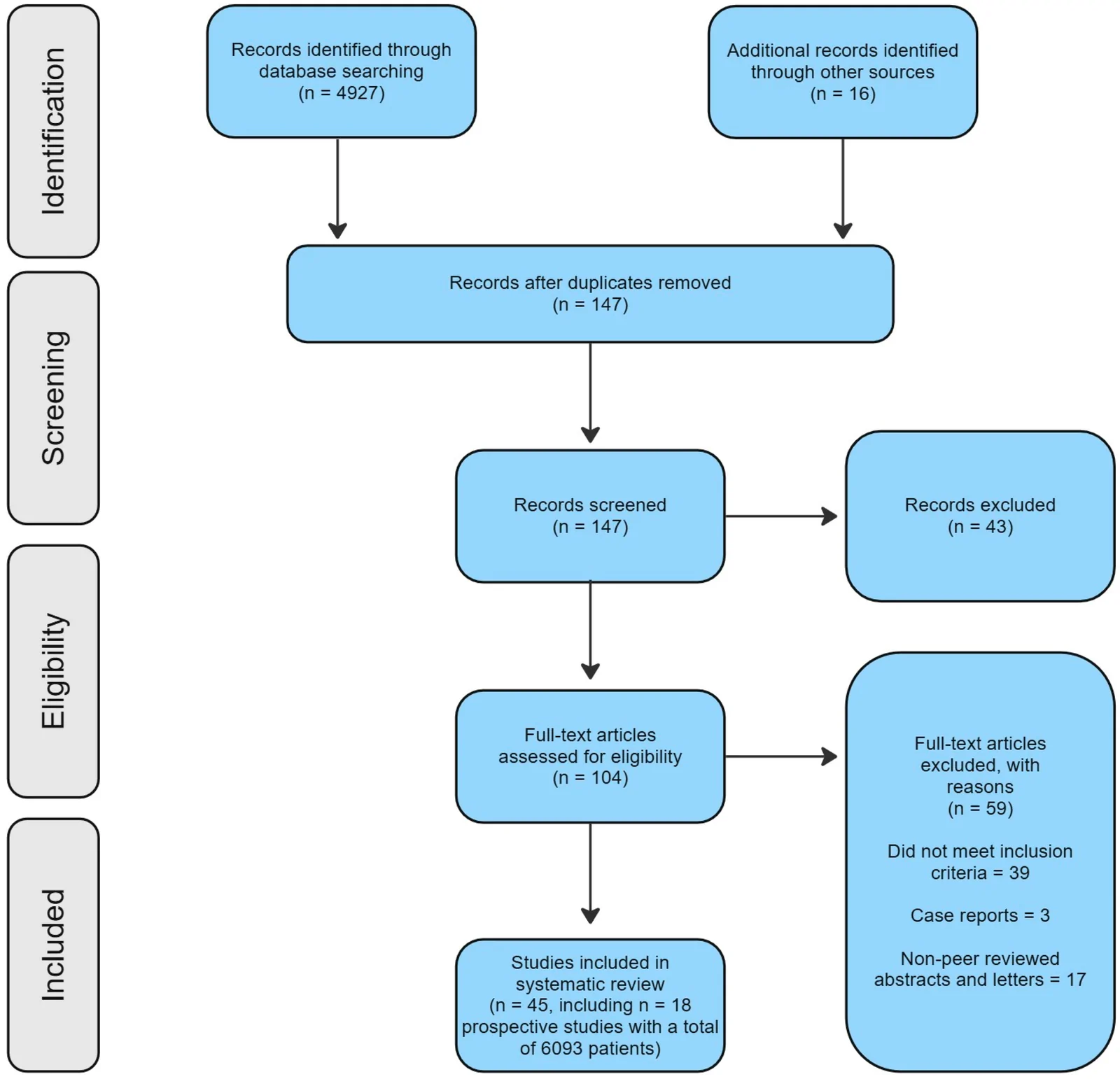
PRISMA flow diagram summarizing the study selection process.

Owing to substantial clinical and methodological heterogeneity across studies—including differences in patient populations, treatment modalities, clearance and recurrence definitions, surveillance intensity, and duration of follow-up—we did not undertake a meta-analysis. In particular, clearance and recurrence were assessed using non-uniform definitions and after substantially different follow-up intervals. Because both outcomes are inherently time-dependent, pooling single-arm proportions across different follow-up durations was considered unlikely to yield a clinically meaningful summary estimate. Findings are therefore presented as a structured qualitative synthesis, with randomized and non-randomized evidence analyzed separately. The study selection process is illustrated in a PRISMA flow diagram (Figure 1). Additional methodological details are provided in the Supplementary Material.

## 3. Results

### 3.1. Study Characteristics and Evidence Landscape

Of 4943 identified records, 45 studies met inclusion criteria, comprising five randomized controlled trials (RCTs) and 40 prospective or retrospective cohort studies within the total of 6093 participants (Figure 1). The evidence base is dominated by PLWH, reflecting the clinical burden of disease in this population, with limited representation of HIV-negative high-risk populations. Across the studies, sample sizes were often small, endpoint definitions inconsistent, surveillance intensity variable, and risk of bias frequently moderate to high. This substantial clinical and methodological heterogeneity limits comparability and precludes firm conclusions regarding the relative effectiveness of different treatment modalities.

### 3.2. RCTs

Randomized evidence for anal HSIL treatment comprises five trials conducted exclusively in PLWH, underscoring the centrality of HIV in this disease context [2,23,24,25,26]. The largest study, ANCHOR (*n* = 4459), demonstrated that HRA-guided treatment of biopsy-confirmed HSIL reduced progression to SCCA compared with active monitoring (hazard ratio 0.43, 95% CI 0.20–0.93) [2]. Electrocautery (EC) was used in 84% of treated participants. Two smaller trials reported higher short-term HSIL clearance with active treatment compared to observation, including infrared coagulation (62% vs. 30% at 12 months) [23] and topical imiquimod (14 vs. 4% after a median follow-up of 33 months) [25].

A three-arm trial comparing EC, imiquimod, and fluorouracil in a mixed AIN population reported complete response (CR) rates of 39%, 24%, and 17%, respectively, with recurrence in 67% of participants within 72 weeks. Only 54–60% of participants had biopsy-confirmed HSIL at baseline, whereas the reported response rates referred to the overall study population [24]. The TREATAIN trial found no significant differences in efficacy between EC, topical cidofovir, and sinecatechins; however, sinecatechins was associated with fewer adverse events [26]. Key characteristics and outcomes are summarized in Table 1. Risk-of-bias assessment using the RoB 2 tool indicated that no trial was at low overall risk of bias; four were rated as having some concerns; and one as high risk (Table 2).

### 3.3. HIV-Related Immunological Parameters in RCTs

HIV-related immunological parameters, including current CD4 count, CD4 nadir, HIV viral load or viral suppression, and antiretroviral therapy status, were reported as baseline characteristics in all randomized trials [2,23,24,25,26]. However, their associations with treatment outcomes were evaluated inconsistently. Most studies found no independent association between current CD4 count, CD4 nadir, or HIV viral load and treatment response. In contrast, the TREATAIN trial identified a CD4 nadir > 200 cells/µL as the only independent predictor of treatment response [26], whereas Richel et al. observed an association between current CD4 count > 500 cells/µL and treatment response only in univariable analysis [24]. Analyses relating HIV-specific immunological parameters to HSIL recurrence were largely absent. Although the TREATAIN trial performed a multivariable analysis of recurrence, no independent predictors were identified [26]. Similarly, the ANCHOR trial evaluated CD4 nadir only in relation to progression to anal cancer and found no significant association [2].

### 3.4. Results Including Non-Randomized Trials

Beyond the five RCTs, 40 non-randomized studies fulfilled the inclusion criteria, comprising 13 prospective observational or pilot studies and 27 retrospective cohort studies. Findings from the prospective studies are summarized in Supplementary Table S4. Most non-randomized studies were small, single-center cohorts with variable definitions of complete response and recurrence, heterogeneous surveillance intervals, and limited follow-up beyond 24 months, thereby limiting assessment of durability.

### 3.5. Ablative Therapies

Electrocautery (EC) is performed once to several times until clearance, is suitable for intra- and perianal lesions, and is relatively inexpensive and quick to administer, which likely explains why it is the most commonly used therapy in the countries of the global west [14,21]. Most data for ablative therapies derive from PLWH populations. EC was the most frequently reported ablative modality and was applied in 84% of treated participants in the ANCHOR trial [2]. Across prospective and retrospective cohorts in predominantly PLWH cohorts, CR rates ranged from 22% to 78% [24,26,27,28,29,30,31]. In the RCT study by Richel et al., EC achieved a CR rate of 39%, compared with 24% for imiquimod and 17% for fluorouracil (*p* = 0.027) [24]. These response rates were reported for the overall study population, which included both LSIL and HSIL, and should therefore not be interpreted as HSIL-specific estimates. In a small prospective cohort of 29 PLWH, 79% developed persistent or recurrent HSIL during follow-up, highlighting limited durability of response [29].

Trichloroacetic acid (TCA) is applied locally under HRA guidance [14,32]. It was evaluated primarily in retrospective series, with CR rates ranging from 28% to 72% [32,33,34]. In an observational, single-center study, response was higher with TCA (73%; 95% CI 67.8–78.5) than with EC (62%; 95% CI 55.3–68.0; *p* = 0.004). Recurrence rates were 28% [32]. Results of the randomized TECAIN trial were published very recently and showed that TCA could be a better tolerated and less complex alternative to EC for the treatment of PLWH (therapeutic success 51% (TCA) vs. 49% (EC) for AIN of any grade 24 weeks after the end of treatment—severe treatment-related pain 4.2% (TCA) vs. 14.8% (EC)). The results of the TECAIN-study are not included in this systematic review because the study was published after January 2026 [35].

Infrared coagulation (IRC) enables intra- and perianal treatment and is a minimally invasive ablative technique typically performed as a single application during anoscopy. IRC was evaluated in several prospective and retrospective cohorts, with CR rates ranging from 3% to 71% [23,36,37,38,39,40].

Radiofrequency ablation (RFA), commonly used for the treatment of Barrett’s esophagus, can also be applied to treat the entire circumference of affected tissue. RFA was evaluated in small cohorts, reporting CR rates of 52–60% at 12 months [41,42,43]. Recurrence was 40% in a prospective observational trial [43].

Surgical excision, typically reserved for selected cases in which carcinoma is suspected, was associated with recurrence rates of 18–30% in retrospective cohorts [44,45]. In a population-based Surveillance, Epidemiology, and End Results (SEER) analysis of 2074 patients with AIN III, 8.2% developed anal cancer after a median of 2.7 years (5-year incidence 9.5%). In contrast, ablative therapies were associated with a significantly reduced cancer risk (OR 0.3, 95% CI 0.1–0.7), suggesting that surgical excision may incompletely address flat or multifocal HSIL, whereas HRA-guided ablation enables more comprehensive lesion control with less toxicity [11].

Overall, across ablative modalities, CR rates, and follow-up varied substantially between studies, with no consistent evidence of durable disease control.

### 3.6. Topical Therapies

Evidence for topical therapies is largely derived from PLWH cohorts.

Imiquimod (5%) is typically self-administered by patients three times weekly for 12 weeks. Across prospective and retrospective cohorts, CR rates ranged from 13% to 79% [24,25,46,47,48,49,50]. In the RCT by Fox et al., CR was 14% (4/28) after a median follow-up of 33 months [25]. In the three-arm trial by Richel et al., CR was 24% in the imiquimod arm, which was significantly lower than in the EC arm (39%; *p* = 0.027). Recurrence was 71% in the imiquimod group, indicating limited durability of response [24].

Topical 5-fluorouracil (5-FU) demonstrated CR rates ranging from 9% to 86% across heterogeneous cohorts [24,51,52,53]. In the RCT by Richel et al., CR in the fluorouracil arm was 17% [24]. Recurrence after response was reported in up to 58% in cohort studies [53], again suggesting limited durability.

Cidofovir was evaluated in prospective cohorts and in the TREATAIN trial. In this RCT, cidofovir achieved an overall response rate of 82% with recurrence reported in 30% by 48 weeks [26]. In contrast, in a phase Ia prospective multicenter trial of 33 participants, CR rates were only 15% and adverse events were reported in 97%, leading to treatment discontinuation in 21% [54]. In a cohort of patients with refractory HSIL, cidofovir was also evaluated with variable response [55].

Sinecatechins, a botanical extract, was also evaluated in the TREATAIN trial. CR or PR of 61%, with a recurrence rate of 36% after 48 weeks was demonstrated. However, adverse events were substantially less frequent with sinecatechins (33% vs. 84%, *p* < 0.001) [26].

Overall, across topical modalities, response rates varied widely, and no consistent evidence of durable disease control was observed.

### 3.7. Other Treatment Modalities

Chemoradiotherapy is the standard treatment for SCCA, providing a biological rationale for the potential efficacy of radiotherapy in anal HSIL [56,57]. However, evidence for radiotherapy in anal HSIL is limited to small retrospective series. [58,59]. Although 5-year local control rates of up to 96% have been reported [58], radiotherapy is associated with a comparatively high treatment burden, potential adverse effects, and may limit subsequent treatment options in the event of invasive recurrence [60]. Further alternative treatment modalities, including carbon dioxide (CO_2_) laser ablation, cryotherapy, argon plasma coagulation (APC), and photodynamic therapy, have been investigated only in small exploratory or retrospective studies with variable response rates [61,62,63,64,65]. Owing to limited sample sizes, heterogeneous study designs, and short follow-up, their role in durable disease control remains undefined. The results of these modalities are summarized in Supplementary Table S5.

### 3.8. Safety and Tolerability

In PLWH, cumulative treatment burden may be amplified due to repeated interventions. Adverse-event (AE) reporting was heterogeneous with respect to definitions, grading, and timing of assessment. Serious adverse events (SAE) were uncommon in randomized trials. In ANCHOR, seven treatment-related SAE (0.3%) occurred in the intervention arm versus one under active monitoring and no SAE were reported after infrared coagulation in the RCT by Goldstone et al. despite a high overall AE rate, highlighting that toxicity was predominantly non-serious [2,23].

Across modalities, adverse events were frequent and predominantly mild to moderate. Toxicity profiles differed between ablative and topical approaches. Ablative therapies were primarily associated with procedure-related symptoms. Pain and bleeding were most common, with rates up to 95% after EC, RFA, or IRC [24,26,32,42,43,61,66]. Topical therapies more commonly induced inflammatory and irritative reactions, including pain, pruritus, urgency, bleeding, and local dermatitis [24,25,26,49,52,54]. Overall AE rates ranged widely, reaching up to 100% in individual cohorts [46]. These findings indicate a high overall treatment burden despite predominantly low-grade toxicity. Reported adverse events in prospective studies are summarized in Table 3.

Direct comparison between modalities is limited by heterogeneous study designs, toxicity definitions, and follow-up schedules. In the TREATAIN trial, sinecatechins was associated with fewer adverse events (33%) compared with EC (97%) and cidofovir (84%) [26]. In the three-arm RCT by Richel et al., grade 3–4 adverse events occurred in 43% of patients receiving imiquimod, 27% receiving fluorouracil, and 18% undergoing EC (*p* = 0.019) [24]. In most studies, symptoms were resolved within weeks, although systematic assessment of cumulative or long-term toxicity was lacking. Available quality-of-life data of the three-arm study by Richel et al. demonstrated clinically relevant impairment across treatment arms, with significant group-by-time interaction effects for pain/discomfort (*p* = 0.001), anxiety/depression (*p* = 0.03), and usual activities (*p* = 0.05), with pain/discomfort rates up to 54% during treatment, anxiety/depression rising to 45% at week 16 after EC, and significantly lower sexual satisfaction in the EC arm at week 16 compared with imiquimod (*p* = 0.01) [24,67]. Quality of life (QoL) data from a small cohort of the ANCHOR trial (*N* = 124) showed that participants randomized to treatment experienced a significant short-term worsening in physical symptoms (mean difference 0.31 ± 0.51; *p* = 0.0001) and psychological functioning (mean difference 0.25 ± 0.64; *p* = 0.022) within the first week after intervention, but returned to rerandomization levels by day 28 [68]. After adjustment for baseline values, improvement in physical symptoms from day 7 to day 28 was greater in the treatment arm compared with active monitoring (*p* = 0.024), while no sustained between-arm differences in overall physical or psychological functioning were observed. These findings suggest that treatment-related QoL impairments are predominantly transient, but cumulative and long-term effects remain insufficiently characterized.

**Table 3:** Reported treatment-related adverse events or symptoms (as reported) in prospective studies of treatment of anal HSIL.

Modality	Trial/Study	Cohort	Reporting (Type), Terminology	SAE	AE/Side Effects	(Tolerability, Duration)	Further Notes
Electrocautery	RCT [2]	2227 (1862 with EC)	Trial related AE and SAE	0	AE: 43 (1.9%) SAE treatment related: 7 (0.3%) Control: AE: 4 (0.2%) SAE: 1 SAE (0.05%) (*p* = 0.07)	QoL (*N* = 124): transient worsening of physical and psychological HRQoL shortly after treatment, with return to baseline by day 28; no sustained between-arm differences [68].	AE reported for entire treatment group (83.6% initial EC); no modality-specific AE rates provided
	RCT [26]	36	Prospective and treatment-related AE assessment; no CTCAE grading	0	AE: 97% (at least 1 AE) Bleeding 78% Pain 72% SAE: 0%	Median duration of AEs: 7 days	Sig. more AE compared to sinecatechins 33% (*p* < 0.001)
	RCT [24]	45	Side-effects graded 1–4 patients reported According to Division of AIDS at the National Institutes of Health.	-	Any side effect: 42 (93%) Grade 1–2: 34 (76%) Bleeding (69%) pain (42%) Grade 3–4: 8 (18%) pain (18%)	3 (7%) stopped because of side-effects AE mostly lasted only a few days after treatment	Difference between the three treatment groups grades 3–4 à *p* = 0.019
	Observational [32]	204 *	Self-reported side effects registered in medical data records	0	Anal discomfort: 175 (86%) Anal pain: 64 (31%) Anal bleeding: 53 (26%) Anal itching: 6 (3%) Anal tenesmus: 5 (3%)	Tolerability: 77%	Sig. more pain and bleeding for EC (*p* = 0.037, *p* = 0.049) and less for itching (*p* < 0.001) compared to TCA
	Observational [66]	83	Uncertain	0	Mild pain: 35 (42%) Limited bleeding: 13 (16%) Local infection: 2 (3%) No SAE after treatment	Pain: lasting <3–5 days 0% interruption due to side effects	
	Observational [29]	29	Telephone survey		uncontrolled pain: 16 (55%) Most of the pain was associated with bowel movements. 21/24 resumed receptive anal intercourse after an average duration of 5.7 months	Uncontrolled pain: lasted for a mean of 2.9 weeks (range, 1 day to 4 months) In 16 pain lasted less than two weeks	Third party performed a telephone survey
Infrared Coagulation	RCT [23]	60	Treatment-related AEs graded 1–5 (no CTCAE specified)	0	AE: 87% (≥1 treatment-related AE: 87%) Grade 1–2: 87% Pain: 48 (80%) Bleeding: 47 (78%) Grade 3–5: 0% SAE: 11 (not treatment related)		
	Observational [36]	124	NR	-	NR	NR	
Radio-frequency Ablation	Observational [43]	49	Prospective AE recording; no CTCAE; severity classified as mild/moderate/ severe.	0	Pain: 41 (85%) (Circumferential RFA) Mild: 31 (63%) Moderate: 10 (20%) Bleeding: 41 (85%) (Circumferential RFA) Mild: 40 (82%) Moderate: 1 (2%) SAE: 1 (one unrelated death)	Pain (mild/median) Median duration 21/31 days (range 4–91) Bleeding (mild/median): Median duration 20/21 days (range 3–87) No long-term functional impairment	AE related to Circumferential RFA
	Observational [42]	21 (with diary)	AE: reported throughout the trial Tolerability: post-RFA diary	0	Diary: Bleeding (defecation): 20 (95%) Bleeding (defecation independent): 12 (57%). moderate: 7 (33%) severe: 1 (5%) Moderate incontinence: 2 (10%) Severe incontinence: 2 (10%) Median peak pain score was 3/10 AE: 4 (19%) with ≥1 AE SAE: 0 (2 SAE non-treatment related)	-Bleeding without bowel movement: lasted median of 2 (0–16) days -severe bleeding resolved within a day without intervention -moderate incontinence: lasted 4 and 7 days -severe incontinence: lasted 1 day -median peak pain score first 5 days post-RFA	
Argon plasma coagulation	Phase II [61]	20 47 treat-ments	Questionnaires immediately after the procedure, by phone at 24 h and 1 week following each treatment, and in person semi-annually for 2 years	0	AE overall at least 84% (from Figure 4 of the cited publication) Pain: median grade of 6/10) 24 h and 1 week post-treatment: 65–70% of (some degree of pain) Bleeding (all minor, only when passing stools): 24 h: Minor 21/46 (46%) One week: 14/47 (30%) of participants after SAE: 0%	At 6 months: 0% at 12 or 18 months: 3 intermittent anal pain one participant ulcer and tenesmus after second treatment, with spontaneous resolution after 1 month three anal or perianal herpes recurrence no long-term side effects	
Surgery	Observational Hidalgo-Tenorio et al. 2021 [45]	47	Uncertain	-	AE: 100% bleeding with defecation: 32 (68%) pain requiring analgesics: 39 (83%) rectal incontinence: 1 (2%) transient anal stenosis: 3 (6%)	Median duration of 15 days post-surgery (IQR: 7–21 days)	Sig. more than with imiquimod (*p* = 0.046))
Carbon Dioxide Laser	Observational [62]	49	Graded according to Division of AIDS Table for Grading the Severity of Adult and Pediatric Adverse Events toxicity table	-	Any recorded AE: 15 (31%) Grade 1: 12 (24.5%) Grade 2: in 3 (6.1%) mainly mild pain or bleeding SAE: 0% 69.4% (*n* = 34) with no symptoms after the procedure.	Average symptom length of 1.9 days. No variables significantly associated with tolerability to treatment	AEs data collected immediately after session and by phone in 1–7 days, or at the follow-up visit.
Trichloroacetic acid	Observational [32]	143 *	Self-reported side effects registered in medical data records	0	Anal discomfort: 113 (79%) Anal pain. 30 (21%) Anal bleeding: 24 (17%) Anal itching: 25 (18%) Anal tenesmus: 6 (4%)	Tolerability: 81%	Sig. less pain and bleeding for TCA (*p* = 0.037, *p* = 0.049) and more itching (*p* < 0.001) compared to EC
Imiquimod	RCT [24]	53	Side-effects graded 1–4, reported by the patient according to Division of AIDS at the National Institutes of Health.	-	Any side effect: 48 (91%) Grade 1–2: 25 (47%) Mainly pain: 20 (38%), Bleeding: 16 (30%) Itching: 8 (15%) Grade 3–4: 23 (43%) Mainly pain: 17 (32%)	5 (9%) stopped because of side-effects Mean duration of AE: 5 weeks	Difference between the three treatment groups grades 3–4 *p* = 0.019
	RCT [25]	28	Patient diary	-	Not reported	1 (4%) patient discontinued because of side-effects	
	Observational [45]			-	AE: 1 (2.7%)	Discontinued in one patient (2.7%)	Sig. less than with mucosectomy (*p* = 0.046)
	Observational [49]	63 (66%)	Questionnaire	-	Local total AE: 25 (40%): Considered moderate: 18 (29%) Considered severe: 7 (11%) pain, itching, irritation and burning sensation Systemic total side effects: 13 (21%) considered severe: 3 (5%)	Tolerability: good: 36 (57%), acceptable:21 (33%) Poor: 6 (10%) discontinued: 6 (10%) extended administration interval: 9 (14%)	10 (16%) of the survey respondents used oral analgesia only 63/95 patients evaluated only 36% HSIL of all patients
	Pilot study [46]	28 (22 for AE)	Clinical observation	0	AE overall: 22 (100%) (compliant *n* = 22) Erythema: 22 (100%) Additional AE: 11/22 (50%) Mild erosions: 7 (32%) Severe erosions: 1 (5%) Influenza like symptoms: 4 (18%)	6 noncompliant, reasons: unwillingness to refrain from sexual intercourse, pain during and after imiquimod application, or depression	*N* = 28 (AE detailed for 22 compliant)
5-Fluorouracil	RCT [24]	48	Side-effects graded 1–4, patients reported According to Division of AIDS at the National Institutes of Health (2004)	-	Any side effect: 44 (92%) Grade 1–2: 31 (65%) Pain: 25 (52%) Urge: 22 (46%) Bleeding 19 (40%) Grade 3–4: 13 (27%) Pain: 7 (15%) Urge: 4 (8%)	2 (4%) of 48 stopped because of side-effects AE lasted mean of 7 weeks	Difference between the three treatment groups grades 3–4 à *p* = 0.019
	Pilot study [52]	46	Prospective and treatment-related AE assessment; no CTCAE grading	**-**	Any side effect: 39 (85%) Mild: 17 (37%) local irritation or minor urge Moderate/strong: 22 (48%) pain, strong urge to defecate, clinical signs of proctitis, local irritation, bleeding	2 discontinued (4 weeks), 5 temporarily interrupted (≤3 weeks) or reduced frequency, 44/46 completed treatment, No long-term toxicity reported	
Cidofovir	RCT [26]	28	Prospective and treatment-related AE assessment; no CTCAE grading	0	AE: 86% (at least 1 AE) Perianal inflammation 36% Itching 43%		Sig. more AE compared to sinecatechins 33% (*p* < 0.001)
	Multicenter pilot trial [54]	33	Diary	0	Overall AE: 32 (97%) Local AEs: Pain/burning/irritation: 76% (all grade 1–2) Ulceration: 39% (grade 1–2) Bleeding: 21% (mild) Systemic AEs: Proteinuria: 21% Anemia: 9% Neutropenia (grade 2): 9% Various mild/moderate infections Grade 3 AEs: 3 (9%) 1 invasive cancer 2 systemic infections (likely unrelated) SAE: 3 (not treatment related, cancer uncertain)	Long-term toxicity Not assessed	Many less frequent mild or moderate AE
Sinecatechins	RCT [26]	36	Prospective and treatment-related AE assessment; no CTCAE grading	0%	AE: 33% (at least 1 AE) Pain: 2.8% Bleeding: 2.8%		Sig. less AE compared to EC and cidofovir (*p* < 0.001)

AE = adverse event; APC = argon plasma coagulation; CTCAE = common terminology criteria for adverse events; EC = electrocautery; HSIL = high-grade squamous intraepithelial lesion; HRQoL = health-related quality of life; IQR = interquartile range; NR = not reported; QoL = quality of life; RCT = randomized controlled trial; RFA = radiofrequency ablation; SAE = serious adverse event; TCA = trichloroacetic acid. When specified, only trial related AE and SAE are reported, * episodes not patients; reporting terminology varied and included “adverse events”, “side effects”, “symptoms”, and diary-based patient-reported outcomes.

### 3.9. Vaccination for Therapy and Tertiary Prevention

RCTs evaluating prophylactic HPV vaccination for secondary prevention of anal HSIL have not demonstrated consistent reduction in recurrence or lesion regression [69,70,71,72]. However, therapeutic HPV vaccination targeting the E6 and E7 oncogenes represents a promising approach in a phase I/II study, with complete clinical and histological regression in 21% (8/38) overall and 40% (4/10) at the highest vaccine dose [73]. Detailed findings from RCTs and meta-analyses are summarized in the Supplementary Material.

## 4. Discussion

The therapeutic landscape of anal HSIL in PLWH has shifted fundamentally following the ANCHOR trial, which provided high-quality evidence that treatment of biopsy-confirmed anal HSIL reduces the risk of progression to SCCA in PLWH [2]. Recent international guidelines consequently recommend evidence-based, risk-adapted screening and treatment strategies for high-risk groups, especially PLWH [13,15]. However, available randomized evidence suggests that durable disease control remains difficult to achieve, as reflected by limited CR and high recurrence rates. Substantial clinical and methodological heterogeneity, including differences in lesion burden, endpoint definitions, and follow-up duration, limits direct comparisons between treatment modalities and the assessment of long-term treatment outcomes. Moreover, only two randomized head-to-head trials provide insufficient evidence to determine the optimal treatment modality. The certainty of the available comparative evidence is further constrained by methodological limitations, as most RCTs were judged to have some concerns regarding risk of bias and one was assessed as having a high risk of bias. In particular, the apparent superiority of EC over 5-FU and imiquimod observed in the trial by Richel et al. should be interpreted cautiously, as response outcomes were reported for the overall study cohort rather than specifically for participants with HSIL. Despite these limitations, EC was the predominant treatment modality in ANCHOR and is currently among the most widely used and best-characterized treatment modalities in expert practice, likely reflecting its wide availability, low cost, ease of application, and predominantly self-limiting adverse events [2,14,68]. Alternative modalities may nevertheless be appropriate in selected cases; for example, topical therapies provide a non-ablative treatment option, whereas IRC offers a superficial ablative approach with minimal bleeding. Accordingly, the field has now entered a second phase focused on defining how durable disease control can be achieved, identifying which patients derive sustained benefit from repeated intervention, and minimizing the cumulative treatment burden. This challenge largely reflects the biological persistence of HPV-associated field disease in PLWH [35].

HIV infection fundamentally alters the natural history of anal HSIL through impaired immune surveillance, persistent HPV infection, and increased field cancerization [74]. These mechanisms likely explain the high recurrence rates observed across treatment modalities and support the concept of anal HSIL in PLWH as a chronic, relapsing condition rather than a discrete lesion amenable to single-intervention cure. Although short-term clearance rates are often favorable, reported recurrence rates commonly ranged from 30% to 70% across modalities and were predominantly driven by metachronous rather than local disease [24,26,42,61,66]. These patterns likely reflect persistent oncogenic anal HPV infection and the biological phenomenon of field carcinogenesis in high-risk populations. Host immune status may further contribute to treatment response and recurrence risk [62]. Although HIV-related immunological parameters were routinely reported at baseline, evidence linking immune status to treatment outcomes remained limited. Where evaluated, immune-related parameters were generally not independently associated with treatment response, with the exception of the TREATAIN trial, in which a CD4 nadir > 200 cells/µL predicted treatment response, and analyses specifically addressing HSIL recurrence were largely lacking [26]. These findings challenge the assumption that repeated lesion-directed therapy alone is sufficient for sustained cancer prevention and highlight the absence of a validated biological risk model to guide treatment intensity and duration. Whether these findings are generalizable to other immunocompromised populations or HIV-negative individuals remains uncertain, as the available evidence is derived predominantly from PLWH [75]. Given the high recurrence rates, strategies to reduce repeated interventions and associated functional impairment are urgently needed; however, alternative therapeutic approaches remain insufficiently studied. Although radiotherapy has demonstrated favorable local control in small retrospective series, current evidence is insufficient to recommend radiotherapy for anal HSIL outside carefully selected, multidisciplinary-evaluated cases with extensive or refractory disease, particularly given its greater treatment burden, higher risk of adverse effects, and substantially greater resource requirements compared with other treatment modalities [14,58].

The benefits of treatment should be considered alongside its cumulative burden. In the ANCHOR trial, treatment was associated with few treatment-related SAEs and no sustained deterioration in health-related quality of life compared with active monitoring, supporting the overall safety and feasibility of contemporary HRA-guided treatment [2,68]. In contrast, smaller studies reported clinically relevant adverse effects, with ablative therapies primarily causing transient pain [24,26,32,42,43,61,66] and topical therapies more frequently inducing inflammatory and irritative reactions [24,32,46,54]. Furthermore, unlike ANCHOR, the RCT by Richel et al. demonstrated clinically relevant, albeit transient, impairment in QoL and treatment discontinuation [24,67]. These findings suggest that treatment-related morbidity should not be underestimated, particularly in patients requiring repeated interventions over time, and is likely influenced by both the treatment modality and operator experience.

Although recent guidelines recommend treatment of biopsy-confirmed anal HSIL not only in PLWH but also in other high-risk populations [14,15], current evidence does not permit reliable biological risk stratification to guide individualized treatment. In particular, it remains unclear which patients derive sustained benefit from repeated intervention and whether biological risk stratification could inform individualized treatment and post-treatment surveillance. Persistent HPV16 has been associated with a lower likelihood of anal HSIL clearance and may therefore help to identify patients at increased risk of persistent disease who may benefit from closer surveillance and risk-adapted management [76]. A systematic review and meta-analysis showed that, as an isolated test, HPV-oncogene mRNA detection performed better than high-risk HPV-DNA, HPV16 genotyping, or p16 detection for screening for anal HSIL/cancer [77]. Host-cell DNA methylation markers have emerged as promising candidates for identifying biologically advanced lesions at increased risk of progression, whereas the prognostic value of currently available tissue biomarkers, including p16, Ki-67, and HPV-E4, appears more limited [78]. Circulating cell-free HPV-DNA has been evaluated for the diagnosis and management of HPV-associated anogenital cancers including SCCA [79]. Possibly, the detection of circulating cell-free HPV-DNA is also suitable for blood-based screening for anal HSIL [80]. However, none of these biomarkers has yet been prospectively validated to guide treatment selection, treatment intensity, or post-treatment surveillance of anal HSIL.

This review has several limitations. First, the protocol was not prospectively registered. Second, the interpretation of the findings was constrained by the quality and heterogeneity of the available evidence. Most included studies were small, non-randomized cohorts with moderate to high risk of bias, and the randomized trials were conducted exclusively in PLWH, limiting generalizability to HIV-negative populations. Clinical and methodological heterogeneity precluded quantitative meta-analysis and limited direct comparisons between treatment modalities. In addition, incomplete reporting of long-term outcomes, cumulative toxicity, and patient-reported outcomes restricted the assessment of treatment durability and overall treatment burden.

## 5. Conclusions

Treatment of biopsy-confirmed anal HSIL in PLWH is now supported by high-quality evidence and represents an important strategy for reducing the risk of progression to SCCA. However, durable disease control remains challenging because of frequent recurrence and the biological persistence of HPV-associated field disease. Current comparative evidence is insufficient to establish the superiority of any single treatment modality, and long-term outcomes remain limited by heterogeneous study designs and the absence of validated biomarkers to guide individualized management. The available evidence supports viewing anal HSIL as a chronic, relapsing disease in PLWH requiring longitudinal management—whether this paradigm applies to HIV-negative individuals or other immune-compromised populations remains uncertain. Future research should prioritize adequately powered comparative trials with standardized outcome definitions, longer follow-up, patient-reported outcomes, and biomarker-guided risk stratification to optimize long-term prevention of SCCA.

## Figures and Tables

**Table 1:** Randomized controlled trials of treatment of anal HSIL in PLWH populations.

**Author/Date**	**Therapy/** **Randomization**	**Primary Endpoint**	**Population**	**Follow Up (Median/Months)**	**Participants** **(N)**	**HSIL (%) ‡**	**CR (%)**	**PR (%)**	**Progression to SCCA (N)**	**Main Results**	**Side Effects (N/%)**	**Overall RoB (RoB 2)**
Palefsky et al. 2022 [2] (ANCHOR)	Various (electro-cautery ablation 84%) RCT 1:1 (AT vs. AM)	Progression to anal cancer	PLWH	25.8	Active therapy *N* = 2227	100	NR	NR	9	Sig. reduced progression to SCC with AT by 57% (*p* = 0.03)	SAE 7 (0.3%) AE 43 (1.9%)	Some concerns
					AM *N* = 2219	100	NR	NR	21		SAE 1 (0.05%) AE 4 (0.2%)	
Goldstone et al. 2019 [23]	Infrared Coagulation Ablation (1–3 Tx) RCT 1:1 (AT vs. AM)	Complete clearance of index lesions (months 12)	PLWH	12	Infrared Coagulation *N* = 60	93	62	20	NR	AT: Sig. more clearance of index lesions (*p* < 0.001), 71% disease-free with AT (12 m)	SAE 0% AE 87%	Some concerns
					AM *N* = 60	92	30	17	NR		SAE 0%/ AE 7%	
Fox et al. 2010 [25]	Imiquimod RCT 1:1 (AT vs. placebo)	Response	PLWH MSM	33	Imiquimod *N* = 28	100	14	29	NR	AT: Positive outcome (clearance/ regression) (*p* = 0.003), disease-free 61% (36 months)	not reported	Some concerns
					Placebo *N* = 25	100	4	0	NR		not reported	
**Author/Date**	**Therapy/** **Randomization**	**Primary Endpoint**	**Population**	**Follow Up (median/months)**	**Participants** **(N)**	**HSIL (%)** ‡	**CR (%)**	**PR (%)**	**Recurrence**	**Main Results**	**Side Effects Quality in %**	**Overall RoB (RoB 2)**
Richel et al. 2013 [24]	Imiquimod vs. Fluorouracil vs. Electro-cautery RCT 1:1:1	Histological resolution of AIN (after 24 weeks)	PLWH MSM	6	Imiquimod *N* = 54	57 †	24 §	11 §	71% (72 weeks)	Electrocautery = more complete response than 5-FU and imiquimod (*p* = 0.027) overall high recurrence rates	Grade 3–4: 43%	Some concerns *
					5-FU *N* = 48	60 †	17 §	13 §	58% (72 weeks)		Grade 3–4: 27%	
					Electrocautery *N* = 46	54 †	39 §	7 §	68% (72 weeks)		Grade 3–4: 18%	
Burgos et al. 2025 [26]	Electrocautery vs. Cidofovir vs. Sinecatechins RCT 1:1:1	Histological resolution of HSIL	PLWH	11	Electrocautery *N* = 36	100	22	47	28% (48 weeks)	Efficacy: No statistically significant difference between treatments (overall response)	AE: 97%	High
					Cidofovir *N* = 28	100	25	57	30% (48 weeks)		AE: 86%	
					Sinecatechins *N* = 36	100	11	50	36% (48 weeks)		AE: 33%	

Abbreviations: AT = Active Therapy; AM = Active Monitoring; RCT = Randomized Controlled Trial; HSIL = High-grade Squamous Intraepithelial Lesion; CR = Complete Response; NR = Not reported; PR = Partial Response; RoB = Risk of Bias; SCCA = Squamous Cell Carcinoma; SAE = Serious Adverse Event(s) (treatment related); AE = Adverse Event(s); PLWH = People Living With HIV; MSM = Men Who Have Sex With Men; 5-FU = 5-Fluorouracil; * High risk of bias for recurrence outcome due to restriction of follow-up to responders; † HSIL; ‡ HSIL (%) indicates the proportion of participants with HSIL in the study population (baseline). § CR and PR refer to the overall study cohort including LSIL.

**Table 2:** Risk of bias assessment of randomized controlled trials (RoB 2).

Study	Randomization Process	Deviations from Intended Interventions	Missing Outcome Data	Measurement of the Outcome	Selection of the Reported Result	Overall Risk of Bias
Palefsky et al., 2022 [2] (ANCHOR)	Some concerns	Some concerns	Low	Low	Low	Some concerns
Goldstone et al., 2019 [23]	Low	Low	Some concerns	Some concerns	Low	Some concerns
Richel et al., 2013 [24]	Some concerns	Low	Some concerns	Low	Low	Some concerns
Fox et al., 2010 [25]	Low	Low	Some concerns	Low	Some concerns	Some concerns
Burgos et al., 2025 [26] (TREATAIN)	Low	High	Some concerns	Some concerns	Low	High

Risk of bias was assessed using the Cochrane Risk of Bias 2 (RoB 2) tool. Judgments were made independently by two reviewers across five domains: bias arising from the randomization process, bias due to deviations from intended interventions, bias due to missing outcome data, bias in measurement of the outcome, and bias in selection of the reported result. Overall risk of bias was classified as low, some concerns, or high according to RoB 2 guidance. High risk of bias was primarily attributable to open-label designs with unblinded outcome assessment and, in one study, restriction of recurrence analyses to responders, introducing post-randomization selection. Some concerns typically reflected open-label designs, incomplete reporting of allocation concealment, modified intention-to-treat analyses, or uncertainty regarding handling of missing data.

## Data Availability

No new data were created in this study. Data were extracted from previously published studies cited in the manuscript. The extracted data are available from the corresponding author upon reasonable request.

## Supplementary Materials

The following supporting information can be downloaded at: https://www.mdpi.com/article/10.3390/cancers18162577/s1, Figure S1: Progression from anal normal squamous epithelium to invasive carcinoma that occurs in PLWH. Persistent high-risk HPV infection drives the stepwise evolution through precancerous lesions—low-grade and high-grade squamous intraepithelial lesions (LSIL and HSIL) — culminating in anal squamous cell carcinoma, as indicated. Figure created with Biorender. Abbreviations: HPV = Human Papillomavirus; Table S1: PRISMA 2020 Checklist; Table S2: Overview of Common Classifications and Terminology in Anal Dysplasia. Table S3: Screening recommendations by the International Anal Neoplasia Society and German-Austrian Guidelines. Table S4: Prospective non-randomized studies of treatment for anal HSIL predominantly in PLWH. Table S5: Summary of selected studies evaluating alternative treatment modalities for anal HSIL. References [81,82,83,84,85,86,87,88,89,90,91,92,93,94,95,96,97,98,99,100,101,102,103,104,105,106,107,108] are cited in the Supplementary Materials.
