# Supplementary material for: Anal High-Grade Squamous Intraepithelial Lesions in People Living with HIV: A Systematic Review of Treatment Outcomes
Source: Cancers (Basel). 2026 Aug 11;18(16):2577. doi: 10.3390/cancers18162577 (PMC13510732; doi:10.3390/cancers18162577)
Supplement: Supplementary file 1 [file cancers-18-02577-s001.zip › cancers-4422320-supplementary.pdf]

Supplementary Material

Table S1 PRISMA 2020 Checklist

Section and Topic	Item #	Checklist item	Location where item is reported
**TITLE**			
Title	1	Identify the report as a systematic review.	Title page
**ABSTRACT**			
Abstract	2	See the PRISMA 2020 for Abstracts checklist.	Abstract
**INTRODUCTION**			
Rationale	3	Describe the rationale for the review in the context of existing knowledge.	Introduction
Objectives	4	Provide an explicit statement of the objective(s) or question(s) the review addresses.	Introduction (last paragraph)
**METHODS**			
Eligibility criteria	5	Specify the inclusion and exclusion criteria for the review and how studies were grouped for the syntheses.	Methods – Eligibility criteria
Information sources	6	Specify all databases, registers, websites, organisations, reference lists and other sources searched or consulted to identify studies. Specify the date when each source was last searched or consulted.	Methods – Data sources and search strategy
Search strategy	7	Present the full search strategies for all databases, registers and websites, including any filters and limits used.	Supplementary material
Selection process	8	Specify the methods used to decide whether a study met the inclusion criteria of the review, including how many reviewers screened each record and each report retrieved, whether they worked independently, and if applicable, details of automation tools used in the process.	Methods – Study selection and data extraction
Data collection process	9	Specify the methods used to collect data from reports, including how many reviewers collected data from each report, whether they worked independently, any processes for obtaining or confirming data from study investigators, and if applicable, details of automation tools used in the process.	Methods – Study selection and data extraction
Data items	10a	List and define all outcomes for which data were sought. Specify whether all results that were compatible with each outcome domain in each	Methods –

Section and Topic	Item #	Checklist item	Location where item is reported
		study were sought (e.g. for all measures, time points, analyses), and if not, the methods used to decide which results to collect.	Study selection and data extraction
	10b	List and define all other variables for which data were sought (e.g. participant and intervention characteristics, funding sources). Describe any assumptions made about any missing or unclear information.	Methods – Study selection and data extraction
Study risk of bias assessment	11	Specify the methods used to assess risk of bias in the included studies, including details of the tool(s) used, how many reviewers assessed each study and whether they worked independently, and if applicable, details of automation tools used in the process.	Methods – Risk of bias assessment (ROB2) (ROBINS-I)
Effect measures	12	Specify for each outcome the effect measure(s) (e.g. risk ratio, mean difference) used in the synthesis or presentation of results.	Not applicable (qualitative synthesis)
Synthesis methods	13a	Describe the processes used to decide which studies were eligible for each synthesis (e.g. tabulating the study intervention characteristics and comparing against the planned groups for each synthesis (item #5)).	Methods – Data synthesis
	13b	Describe any methods required to prepare the data for presentation or synthesis, such as handling of missing summary statistics, or data conversions.	Methods – Data synthesis
	13c	Describe any methods used to tabulate or visually display results of individual studies and syntheses.	Methods – Data synthesis
	13d	Describe any methods used to synthesize results and provide a rationale for the choice(s). If meta-analysis was performed, describe the model(s), method(s) to identify the presence and extent of statistical heterogeneity, and software package(s) used.	Not applicable (no meta-analysis performed)
	13e	Describe any methods used to explore possible causes of heterogeneity among study results (e.g. subgroup analysis, meta-regression).	Not applicable (no meta-analysis performed)
	13f	Describe any sensitivity analyses conducted to assess robustness of the synthesized results.	Not applicable (no meta-analysis performed)
Reporting bias assessment	14	Describe any methods used to assess risk of bias due to missing results in a synthesis (arising from reporting biases).	Not performed

Section and Topic	Item #	Checklist item	Location where item is reported
Certainty assessment	15	Describe any methods used to assess certainty (or confidence) in the body of evidence for an outcome.	Not performed
**RESULTS**			
Study selection	16a	Describe the results of the search and selection process, from the number of records identified in the search to the number of studies included in the review, ideally using a flow diagram.	Results – Study characteristics and evidence landscape; Figure 1
	16b	Cite studies that might appear to meet the inclusion criteria, but which were excluded, and explain why they were excluded.	Not reported
Study characteristics	17	Cite each included study and present its characteristics.	Results – Study characteristics; Table 1, Supplements Table S3
Risk of bias in studies	18	Present assessments of risk of bias for each included study.	Results Table 2, Supplements Table S3
Results of individual studies	19	For all outcomes, present, for each study: (a) summary statistics for each group (where appropriate) and (b) an effect estimate and its precision (e.g. confidence/credible interval), ideally using structured tables or plots.	Results – Randomized controlled trials; Tables 1–3
Results of syntheses	20a	For each synthesis, briefly summarise the characteristics and risk of bias among contributing studies.	Results
	20b	Present results of all statistical syntheses conducted. If meta-analysis was done, present for each the summary estimate and its precision (e.g. confidence/credible interval) and measures of statistical heterogeneity. If comparing groups, describe the direction of the effect.	Not applicable (no meta-analysis performed)
	20c	Present results of all investigations of possible causes of heterogeneity among study results.	Not applicable (no meta-analysis performed)
	20d	Present results of all sensitivity analyses conducted to assess the robustness of the synthesized results.	Not applicable (no meta-

Section and Topic	Item #	Checklist item	Location where item is reported
			analysis performed)
Reporting biases	21	Present assessments of risk of bias due to missing results (arising from reporting biases) for each synthesis assessed.	Not assessed
Certainty of evidence	22	Present assessments of certainty (or confidence) in the body of evidence for each outcome assessed.	Not assessed
**DISCUSSION**			
Discussion	23a	Provide a general interpretation of the results in the context of other evidence.	Discussion
	23b	Discuss any limitations of the evidence included in the review.	Discussion
	23c	Discuss any limitations of the review processes used.	Discussion
	23d	Discuss implications of the results for practice, policy, and future research.	Discussion
**OTHER INFORMATION**			
Registration and protocol	24a	Provide registration information for the review, including register name and registration number, or state that the review was not registered.	Not registered
	24b	Indicate where the review protocol can be accessed, or state that a protocol was not prepared.	Not registered
	24c	Describe and explain any amendments to information provided at registration or in the protocol.	Not applicable
Support	25	Describe sources of financial or non-financial support for the review, and the role of the funders or sponsors in the review.	Declarations – Funding
Competing interests	26	Declare any competing interests of review authors.	Declarations – Competing interests
Availability of data, code and other materials	27	Report which of the following are publicly available and where they can be found: template data collection forms; data extracted from included studies; data used for all analyses; analytic code; any other materials used in the review.	Declarations – Data availability

From: Page MJ, McKenzie JE, Bossuyt PM, Boutron I, Hoffmann TC, Mulrow CD, et al. The PRISMA 2020 statement: an updated guideline for reporting systematic reviews. BMJ 2021;372:n71. doi: 10.1136/bmj.n71

EXTENDED BACKGROUND INFORMATION

TERMINOLOGY AND CLASSIFICATION

Given the central role of HIV in HSIL pathogenesis and progression, extended background information is provided with emphasis on PLWH. Historically, anal precursor lesions have been classified using multiple cytological and histopathological systems adapted from cervical pathology, including Bethesda-based cytology and earlier AIN/ASIL grading schemes [1,2].

The Lower Anogenital Squamous Terminology (LAST) guidelines advocated a unified histopathological nomenclature for HPV-associated preinvasive squamous lesions of the lower anogenital tract and recommended standardizing diagnostic terminology to a two-tier LSIL/HSIL system aligned with cytological classification. For lesions previously classified as AIN2, adjunct p16 immunohistochemistry is recommended to support lesion stratification [3], with diffuse "block-type" staining supporting classification as HSIL and p16-negative lesions designated as LSIL [4]. For the present review, we consistently use the LSIL/HSIL terminology to ensure comparability of treatment outcomes across heterogeneous studies. Where studies employed alternative terminology (e.g., AIN), original terms were retained if a clear translation to HSIL was not feasible. The non-standard umbrella term "dysplasia" is avoided throughout to maintain terminological consistency. **Table S2** summarizes historical and contemporary classification systems for anal precursor lesions, adapted from the German–Austrian S2k guideline [5].

Lesions were classified according to the LAST (Lower Anogenital Squamous Terminology) framework. Where older or heterogeneous terminology (e.g., AIN 2/3) was used, high-grade lesions were categorized as HSIL based on the histological grading reported in the original studies, provided a clear and unambiguous translation was possible.

Table S2. Overview of Common Classifications and Terminology in Anal Dysplasia

Grade of Dysplasia	Cytological Grading of Anal Dysplasia according to Bethesda [2]	Histopathological Grading of Anal Dysplasia according to LAST [4]		Alternative Terminology	Host-cell Methylation Marker Positivity (ZNF582, ASCL1, SST) [6]	E6/E7 mRNA positive [7,8]
Undetermined	ASC-US					48-80%
	ASC-H					
Negative	NILM	Normal			Negative	23-39%
Little to no dysplasia	LSIL	Condylomas		LGAIN		68-86%
Mild dysplasia	LSIL	AIN 1			⇔	
Moderate dysplasia	LSIL	AIN 2, p16 negative		HGAIN/AIN2+	↑	
High-grade dysplasia / CIS	HSIL	AIN 2, p16 positive			↑↑	95-100%
	HSIL	AIN 3				
Superficial (minimally invasive) carcinoma	CA	SISCCA			↑↑↑	100%
Invasive carcinoma	CA	CA				

*Adapted from the German-Austrian Guideline on Anal Dysplasia and Anal Cancer Screening in People living with HIV (S2k) [5].

Abbreviations: ASC-US = Atypical Squamous Cells of Undetermined Significance; ASC-H = Atypical Squamous Cells, cannot exclude High-grade lesion; NILM = Negative for Intraepithelial Lesion or Malignancy; LSIL = Low-grade Squamous Intraepithelial Lesion; HSIL = High-grade Squamous Intraepithelial Lesion; AIN = Anal Intraepithelial Neoplasia; LGAIN = Low-grade Anal Intraepithelial Neoplasia; HGAIN = High-grade Anal Intraepithelial Neoplasia; p16 = Cyclin-dependent kinase inhibitor 2A (used as a surrogate marker for transforming HPV infections); CIS = Carcinoma in situ; CA = Carcinoma; SISCCA = Superficially Invasive Squamous Cell Carcinoma of the Anus; E6/E7 mRNA = Messenger RNA of HPV oncogenes E6 and E7; ZNF582, ASCL1, SST = Host-cell methylation markers

PATHOGENESIS AND BIOLOGICAL RISK FACTORS

The pathogenesis of anal high-grade squamous intraepithelial lesions (HSIL) is driven by persistent infection with high-risk human papillomavirus (HPV), leading to architectural and cellular abnormalities of the anal epithelium, particularly within the anal transitional zone at the dentate line (**Figure S1**) [9,10]. Sustained expression of the viral oncogenes E6 and E7 disrupts key tumor suppressor pathways, including p53 and retinoblastoma protein, resulting

in deregulated cell-cycle control and accumulation of genetic damage [11,12]. Overexpression of p16 serves as a surrogate marker of transforming HPV infection and reflects functional inactivation of the retinoblastoma pathway in HPV-associated lesions [13]. Diffuse "block-type" p16 expression is characteristic of transforming disease and supports classification as HSIL, whereas p16-negative lesions are considered non-transforming [3,4]. Importantly, p16 is a cellular marker of transforming HPV activity and should not be confused with HPV16 itself. Biological risk factors for HSIL development and persistence include impaired immune surveillance, persistent infection with oncogenic HPV types, and anatomical susceptibility of the anal transitional zone that are particularly relevant to PLWH. These mechanisms parallel the pathogenesis of cervical intraepithelial neoplasia, although differences in anatomy, local immune environment, and hormonal influences may contribute to distinct disease behavior between anal and cervical lesions [10,14].

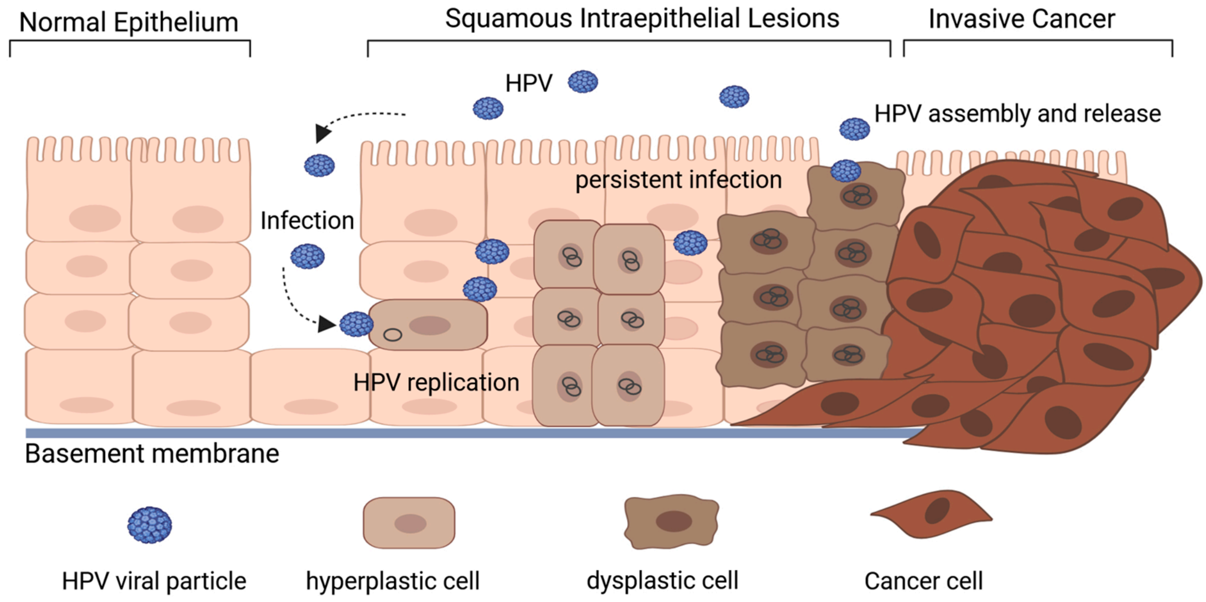

Figure S1. Progression from anal normal squamous epithelium to invasive carcinoma that occurs in PLWH. Persistent high-risk HPV infection drives the stepwise evolution through precancerous lesions—low-grade and high-grade squamous intraepithelial lesions (LSIL and HSIL) — culminating in anal squamous cell carcinoma, as indicated. Figure created with Biorender. Abbreviations: HPV = Human Papillomavirus

PROGRESSION TO CANCER

Anal HSIL is associated with a measurable risk of progression to SCCA, especially in high-risk populations including PLWH [15]. An analysis of the SEER registry (1973–2014) including 2,074 patients with AIN 3 (HSIL) showed that 8.2% progressed to SCCA within a median of 2.7 years; 30.4% were diagnosed at stage ≥cT2. Initial ablative treatment was associated with a

significantly reduced risk of progression (odds ratio [OR] 0.3), and the 5-year cumulative incidence was 9.5% (1.9% per year) [16]. A Danish registry study of 1,222 AIN 3 patients followed for over 12,000 person-years reported a 7.9% progression rate, with a markedly increased risk in individuals living with HIV, particularly those with CD4 counts ≤200 cells/µL (hazard ratio [HR] 4.25) [17]. In a Spanish prospective cohort of 16,274 treatment-naive, HIV-infected adults, 22 SCCA cases were identified (22.3 per 100,000 person-years), with 34.6% occurring in individuals under age 35 [18]. Among PLWH with a prior AIDS diagnosis, the 5-year cumulative incidence of SCCA was 3.4% in females and 3.7% in males [19].

Evidence from post-treatment follow-up is provided by the Anal Cancer/HSIL Outcomes Research (ANCHOR) trial, which demonstrated that treatment of HSIL significantly reduces progression to anal squamous cell carcinoma in PLWH. During a median follow-up of 25.8 months, 9 cases of SCCA occurred in the treatment group (173 per 100,000 person-years) compared with 21 cases in the active monitoring group [20]. Lesion extent was a key determinant of progression, with substantially higher rates observed in individuals with extensive anal involvement. In addition, two small prospective studies in people living with HIV reported progression rates from predominantly high-grade HSIL to anal squamous cell carcinoma of 8.5% and 11% within 60 months, respectively [21,22].

DIAGNOSTICS AND SCREENING

Diagnostic and screening strategies for anal HSIL are highly relevant in high risk populations including PLWH but vary substantially across clinical settings, reflecting limited high-quality evidence and restricted availability of specialized diagnostic procedures [23]. No universally accepted screening strategy has been established. However, emerging longitudinal data from structured high-resolution anoscopy (HRA) programs increasingly inform detection rates, progression dynamics, and risk stratification in high-risk populations. Accordingly, this review does not aim to provide screening recommendations but to contextualize diagnostic approaches relevant to HSIL detection and therapeutic management [24].

Anal cytology, sometimes complemented by digital rectal examination, is commonly used as an initial approach in high-risk populations (particularly people living with HIV), but performance is variable: in a systematic review and meta-analysis, sensitivities for LSIL/HSIL ranged from 44–89%, and specificity for HSIL from 33–94%, depending on thresholds and study design [25,26]. This approach is reflected in several clinical guidelines and practice

parameters [27,28]. High-resolution anoscopy (HRA) with targeted biopsy remains the reference standard for HSIL diagnosis. However, its availability is limited and diagnostic accuracy is strongly influenced by examiner expertise [29–31]. Even in structured care pathways, completion of recommended treatment and follow-up after diagnosis of anal precancerous lesions remains suboptimal [32]. High-risk HPV testing using HPV DNA and/or E6/E7 mRNA assays is increasingly incorporated as a triage or adjunctive tool, particularly in settings with limited HRA capacity [33]. In modelling analyses of MSM living with HIV aged ≥35 years, preventing one case of anal cancer required 922 annual cytology screens, 331 high-resolution anoscopies, and 153 treatments of high-grade lesions; these effect sizes are of a magnitude broadly comparable to those observed in established colorectal cancer screening and prevention programs, underscoring the potential population-level impact despite the relatively low absolute event numbers [20]. Recent international guidelines, including those from the International Anal Neoplasia Society and the German–Austrian S2k committee, propose risk-adapted diagnostic pathways rather than population-wide screening; detailed recommendations and algorithms are provided in **Table S3** [5,33].

Table S3. Screening recommendations by the International Anal Neoplasia Society and German-Austrian Guidelines

**Population**		**International Anal Neoplasia Society (IANS)**[33]	**German-Austrian Guideline**
**very high risk** (category A[1] or "should be offered"[3])	Age-related	• MSM with HIV from the age of 35<br>• Transgender women with HIV from the age of 35<br>• Women with HIV from the age of 45<br>• MSW with HIV from the age of 45<br>• MSM and TGW not with HIV from the age of 45	• MSM with HIV from the age of 35<br>• Transgender women with HIV from the age of 35<br>• All individuals with HIV from the age of 45<br>• MSM and TGW without HIV from the age of 45
	Not -age-related	• History of vulvar HSIL or cancer within 1 year of diagnosis<br>• People with ≥10 years after organ transplantation	• People with HIV and high-grade cervical, vulvar, or penile dysplasia<br>• People with HIV with a CD4+ T-lymphocyte nadir <200/µl.<br>• women with VIN III<br>• People ≥10 years after organ transplantation

**high risk** (category B[2] or "should be considered"[3])	Age-related	• Cervical/vaginal cancer from the age of 45<br>• Cervical/vaginal HSIL from the age of 45<br>• Perianal warts (male or female) from the age of 45<br>• Persistent cervical HPV16 (>1 year) from the age of 45<br>• Other immunosuppression (e.g., Rheumatoid arthritis, Lupus erythematosus, Crohn's disease, Ulcerative colitis, systemic steroid therapy) from the age of 45	
	Not age-related		• people <10 years after organ transplantation or with other long-term immunosuppressive therapy<br>• Women with persistence of cervical HPV16 and/or after CIN-III<br>• People with persisting anogenital warts
**Interval**	months	12-24 (depending on type of test)	12-24 (using algorithm)

[1] IANS: Risk Category A (incidence ≥10-fold compared to the general population)
[2] IANS: Risk Category B (incidence less than 10-fold higher than the general population)
[3] German-Austrian: the term "should" is used to translate both the German "soll" and "sollte," with the latter indicating a lower degree of urgency or recommendation strength by adding "should be considered", reflecting its subjunctive nuance.

Abbreviations: IANS = International Anal Neoplasia Society; MSM = Men Who Have Sex with Men; MSW = Men Who Have Sex with Women; TGW = Transgender Women; HIV = Human Immunodeficiency Virus; HSIL = High-Grade Squamous Intraepithelial Lesion; VIN = Vulvar Intraepithelial Neoplasia; CD4+ = Cluster of Differentiation 4 Positive T-Lymphocytes; µl = Microliter; HPV = Human Papillomavirus; CIN = Cervical Intraepithelial Neoplasia

PROPHYLACTIC VACCINATION TO PREVENT ANAL HSIL AND ANAL CANCER

Prophylactic HPV vaccination was initially developed to prevent cervical cancer and has demonstrated efficacy in reducing anal HPV infection and anal intraepithelial neoplasia (AIN), particularly when administered prior to HPV exposure. A systematic review and meta-analysis of randomized controlled trials and real-world studies demonstrated high vaccine efficacy against anal HPV infection and AIN in HIV-negative individuals vaccinated at ≤26 years, with pooled vaccine efficacy estimates of 84% in per-protocol populations, 55% in intention-to-treat populations, and 77% in real-world settings; effectiveness declined with increasing age at vaccination and was attenuated among PLWH [34]. In a randomized, double-blind trial including 602 HIV-negative MSM aged 16–26 years, the quadrivalent HPV vaccine reduced the incidence of AIN caused by vaccine HPV types (HPV-6, -11, -16, -18) by 50.3% (95% CI 25.7–67.2) in the intention-to-treat population and by 77.5% (95% CI 39.6–93.3) in the per-protocol population. For AIN associated with any HPV type, vaccine efficacy was lower but remained measurable [35]. Long-term follow-up of this randomized cohort demonstrated durable protection for up to 10 years. In the early vaccination per-protocol population, no vaccine-type external genital lesions were observed during follow-up, and the incidence of vaccine-type anal intraepithelial neoplasia or anal cancer in MSM was markedly lower than in the placebo group during the base study (20.5 vs. 906.2 per 10,000 person-years), with similar protective trends observed in catch-up vaccination group [36]. Immunogenicity studies in adults have shown that prophylactic HPV vaccination induces robust antibody responses, while clinical protection is largely confined to HPV-naïve individuals, underscoring the importance of early vaccination [37]. Population-level data further support the effectiveness of early vaccination. In countries with high vaccine uptake, national HPV vaccination programs have led to marked reductions in HPV-related disease and substantial herd immunity effects. Modeling studies suggest that high coverage with the nonavalent HPV vaccine could prevent up to 90% of cervical cancers and approximately 96% of anal squamous cell carcinomas [38]. In a large Danish nationwide cohort including more than 968,000 women, HPV vaccination before age 17 was associated with a 70% reduction in the risk of AIN3 or anal cancer, whereas vaccination between ages 17 and 32 showed no measurable protective effect against anal HSIL [39]. Importantly, prophylactic HPV vaccination prevents new anal HPV infections but does not treat established HSIL [40] and should therefore be regarded as a complementary preventive strategy rather than a therapeutic intervention.

VACCINATION FOR THERAPY AND TERTIARY PREVENTION OF HSIL

Early studies on therapeutic vaccines for anal dysplasia yielded unsatisfactory results as over 75% of HIV-negative MSM and 66% of MSM living with HIV showed persistent AIN lesions following therapeutic HPV vaccination [41,42]. While the quadrivalent HPV vaccine (qHPV, Gardasil-4, MSD) was approved as a prophylactic vaccine, some studies have explored its potential therapeutic effects. A randomized, double-blind controlled trial tested three doses of qHPV vs. placebo at 0, 2, and 6 months in 126 MSM living with HIV who had had successfully treated anal HSIL within the last year. The primary outcome was biopsy-proven anal HSIL recurrence at 18 months. No difference was found (P = 0.38) in cumulative HSIL recurrence rates between the qHPV (44/64, 68.8%) and placebo group (38/62, 61.3%) in the intention to treat (ITT) analysis [absolute risk reduction -7.5 (95% confidence interval (CI) -24.1 to 9.2)] despite adequate serological responses to qHPV vaccination [43]. A phase 3, double-blind, randomized, placebo-controlled trial investigated the efficacy of the qHPV in adults living with HIV aged 27 years and older (n=575), evaluating persistent anal HPV infection, anal HSIL, and oral HPV infection. The trial was stopped early by the data and safety monitoring board due to futility. Vaccine efficacy was 22% (95% CI -31 to 53%) for preventing persistent anal infection and 0% (95% CI -44 to 31%) for improving biopsy-proven HSIL outcomes. The only statistically significant efficacy result was 88% (95% CI 2 to 98%) for preventing persistent oral HPV infection, but results were not significant for six-months persistent oral infection including single detection at the final visit (efficacy 32% (95% CI -80 to 74%)) [40]. In a randomized, double-blind, placebo-controlled study with 129 MSM living with HIV, the group who had received qHPV did not differ from the placebo group in terms of anal HSIL (14.1 vs. 13.1%, p = 0.98) or external anogenital lesions (11.1 vs. 6.8%, p = 0.4) during follow-up [44]. Finally, a meta-analysis of 22 studies investigated whether HPV vaccination reduces recurrence of HPV-related disease after treatment of preinvasive genital lesions. Although HPV vaccination significantly lowered the recurrence of cervical intraepithelial neoplasia (CIN2+), particularly with HPV16/18, evidence for reducing recurrence of AIN or other non-cervical HPV-related lesions was inconclusive due to limited studies and participant numbers [45]. Possibly therapeutic vaccines with antigens other than the major HPV capsid antigen L1 could be more promising in the future. A recent phase I/II study investigated a synthetic long peptide vaccine consisting of 13 HPV16 E6- and E7-oncogene peptides in 40 MSM living with HIV who had HPV16-positive high-grade AIN. In the ITT-analysis, 21% (8/38) had complete

clinical and histological regression, and in the group who had received the highest vaccine dose 40% (4/10) had complete regression [46].

METHODS

Data sources and search strategy - full electronic search strategy

The electronic search strategy was developed using a Boolean approach combining terms related to anal high-grade squamous intraepithelial lesions and therapeutic interventions. The following search structure was applied in PubMed, EMBASE, and the Cochrane Library:

("anal intraepithelial neoplasia" OR "AIN" OR "anal HSIL" OR "high-grade anal intraepithelial neoplasia" OR "HGAIN" OR "anal squamous intraepithelial lesion" OR "SIL" OR "anal dysplasia")
AND
(“HIV” OR “PLWH” OR "therapy" OR "treatment" OR "management" OR "local ablative" OR "surgical excision" OR "radiofrequency ablation" OR "infrared coagulation" OR "electrocautery" OR "cryotherapy" OR "laser therapy" OR "argon plasma coagulation" OR "photodynamic therapy" OR "topical therapy" OR "imiquimod" OR "5-fluorouracil" OR "5-FU" OR "sinecatechins" OR "trichloroacetic acid" OR "HPV" OR "human papillomavirus" OR "vaccination" OR "radiation therapy").

Titles and abstracts were screened against predefined eligibility criteria, followed by full-text review. Duplicate publications were identified and excluded. Reference lists of included studies, contemporary clinical guidelines, and clinical trial registries were examined to ensure completeness. Two reviewers independently performed study selection and data extraction. Disagreements were resolved by discussion.

Risk of bias was assessed using the Cochrane Risk of Bias 2 tool for randomized trials and ROBINS-I for non-randomized studies. Detailed assessments are provided in **Table 2**.

Table S4. Prospective non-randomized studies of treatment for anal HSIL predominantly in PLWH

Author / Date	Study type	Population	Therapy	Participants (N)*	Follow up (median/ month)	HSIL (%)	CR (%)	PR (%)	Recur-rence (%)	Main results	ROBINS-I (risk of bias)
Burgos et al. 2024 [47]	Observational single-center study	HIV+	Trichloroacetic acid (2-4 treatments)	(260 episodes)	24 (for recurrence)	100	34	39	28	Remission evaluation after first approach<br><br>Sig. more complete response with trichloroacetic acid (p=0.026)	
			Electrocautery (2-4 treatments)	(227 episodes)	24 (for recurrence	100	30	32	36		
Goldstone et al. 2023 [48]	Prospective, dual-center trial	HIV+ (33%)<br><br>Male (94%)	Radiofrequency ablation (Circumferential) (58% had ≥3 lesions treated)	48 (evaluable)	17.7	100	-	-	40	19% had 1, 15% had 2, 6% had 3 recurrences<br><br>no progression to SCC	Moderate
Fuertes 2022 [49]	Exploratory, pilot, single-arm study	HIV +<br><br>max ≤3 lesions	Carbon Dioxide Laser	48 (PP)	12	100	50	21	29%	71% overall response rate	Serious
Hidalgo-Tenorio et al. 2021 [50]	Longitudinal observational (naturalistic) prospective study	HIV+<br><br>MSM	Mucosectomy<br><br>(31 one, 9 two, 1 four interventions)	47 (+1 after Imiquimod)	36	100	73 (to first line, n=45)	-	15	- Sig. more response rate with Imiquimod<br>- Sig. more retreatment and AEs in surgery group<br>- disease-free period: 36 month (mean) for Imiquimod vs. 48 (median) months for surgery<br>- Retreatment needed: 11 in surgery, 1 in Imiquimod group	Critical
			Imiquimod<br><br>(3 times weekly for 16 weeks), previous surgery in 13.5%;	37 (inclusion of 5 after surgery)			97 (to first line)	-	-		
Fuertes et. al. 2019 [51]	Prospectively followed cohort (Retrospective analysis)	HIV+<br><br>MSM (95%)<br><br>intraanal	Imiquimod<br><br>(15 of 95 included patients with simultaneous therapy)	29 (only ITT HSIL N=29)	5	36	3 (only ITT HSIL N=29)	38 (only ITT HSIL N=29)	-	Imiquimod effective and with overall good tolerability,<br>Less effective than extraanal and Condylomata	Serious

Pokomandy et al. 2018 [52]	Prospective phase II, pilot study	HIV+<br><br>MSM	Argon plasma coagulation<br>1-4 treatments	20	24	100	65%	45%<br>(after 1. treatment)	85%<br>(after 1. treatment including persistence)	Overall succesfull, but with high recurrence rates	Moderate
Goldstone et al. 2017 [53]	Prospective, non-randomized open-label pilot study	HIV-<br><br>≤ half the anal canal circum-ference	Radiofrequency ablation<br>(hemi-circumferential)<br><br>(1-2 treatments)	20	12	100	76<br><br>(lesion-free at ETZ estimated)	-	29<br><br>(Inside ETZ)<br><br>48<br>(anywhere within 1 year)	Primary “endpoint”: HSIL-free survival at 1-year: 74%<br><br>7 (33% with metachronous lesions within 1 year)<br><br>Treatment learning curve	Moderate
Burgos et al. 2016 [54]	Observational cohort study	HIV+<br><br>MSM	Electrocautery<br>(HRA guided)<br><br>(2-4 treatments until clearance)	83	12.1 (mean)	100	33	34	25%	Recurrence (N=14): 10 on ETZ, 4 metachronously<br><br>effectiveness of 113 evaluable cycles of treatment: CR = 24%	Serious
Stier et al. 2013 [55]	Prospective multicentre, open-label, pilot trial	HIV+<br><br>PAIN	Cidofovir 1%<br>(6 treatment cycles, each 14 days)	33<br>(at least one cycle of treatment)	4.5	100	15	36	-	progression to cancer: 1 (3 %)<br><br>Response rates (per-protocol, n=26): CR= 19%; PR=46%; SD=31%; PD = 8%.	Serious
Weis et al. 2012 [56]	Prospective cohort study	HIV+	infrared coagulation<br>(102 were treated, 82 immediately, 20 delayed)	98<br>(treated for HSIL)	15	100	3	71	-	- primary endpoint: At final evaluation, 85/98 (87%) free of HGAIN<br>sig. more remission in treated patients	Serious
			Not treated (NT)<br>(42 were not treated)	42<br>(20 with delayed treatment)	20.3	100	0	7	-	- progression to SCC: 2 (5 %)<br>- residual HGAIN: 37/42 (88%)	
Richel et al. 2010 [57]	Prospective, non-randomized	HIV+<br>MSM<br><br>intraanal	5-Fluorouracil<br>(twice weekly, total of 16 weeks treatment)	46	6<br>(recurrence)	74<br>(AIN 2+3)	35<br>(AIN2+3)<br><br>39	24<br>(AIN2+3)<br><br>17	33<br>(AIN2+3)<br><br>50	76% initially multifocal lesions	Serious

	open-label pilot study	AIN 1–3					(ITT)	(ITT)	(all AIN)		
Wieland et al. 2006 [58]	Prospective, non-randomized open-label pilot study	HIV+ MSM	Imiquimod 5% (3 times a week for 16 weeks)	28	7 (median)<br><br>9.5 (mean)	68	61 (ITT)<br><br>77 (per protocol)	-	17 (among responders ≥5 months follow-up))	Only 22/28 per protocol:<br>Complete clearance end of treatment of N=22:<br>- AIN I: 6/8 (75%)<br>- AIN II: 4/4 (100%)<br>- AIN III: 7/10 (70%)<br>At last follow-up:<br>14/17 with complete response	Serious
Chang et al. 2002 [59]	Prospective non-randomized open label pilot study	Men (100 %)<br><br>HIV+ (78%)<br><br>HIV- (22%)	incisional biopsy + electrocautery (HRA) for HIV+	29	28.6 (mean for HIV+)	100	-	-	79 (HIV+) (persistent/ recurrent)	sig. more recurrence/persistence in HIV+ patients<br><br>Recurrence includes persistent and metachronous lesions.	Serious
				8	32.3 (mean for HIV-)	100			0 (HIV-) (persistent/ recurrent)		

*available for treatment evaluation

Abbreviations: HSIL = High-Grade Squamous Intraepithelial Lesion; CR = Complete Response; PR = Partial Response; ITT = Intention-to-Treat Population; AE = Adverse Event; SAE = Serious Adverse Event; HIV+ = Human Immunodeficiency Virus Positive; MSM = Men Who Have Sex With Men; AIN = Anal Intraepithelial Neoplasia; ETZ = Eligible Treatment Zone; HRA = High-Resolution Anoscopy; PAIN = Perianal Intraepithelial Neoplasia; PD = Progressive Disease; SD = Stable Disease; 5-FU = 5-Fluorouracil, PP = per protocol), recurrence is related to responders if not specified

Table S5. Summary of selected studies evaluating alternative treatment modalities for anal HSIL

Treatment modality	Selected studies	Study design	Population	Treatment outcome	Main limitations
Radiotherapy	Howard et al. [60]	Retrospective series	Perianal AIN 3, n = 31	• Actuarial 5 year local control: 96%<br>• Actuarial 10 year local control: 51%<br>• 3 treatment breaks due to acute RT reaction	Small retrospective serie; heterogeneous treatment regimens
	Ortholan et al. [61]	Retrospective multicenter survey	Mixed (Tis and T1 anal canal carcinoma <1 cm, n = 69	• Initial local control: 99% (68/69)<br>• 5-year disease-free survival: 89%<br>• 6 local recurrences	Included invasive carcinoma rather than isolated HSIL; heterogeneous treatment regimens
Carbon dioxide (CO_2) laser ablation	Fuertes et al. [49]	Prospective single-arm pilot trial	PLWH, ≤3 untreated intra-anal HSILs, n = 52, lesions = 72	• Complete response: 50%<br>• Partial response: 20.8%<br>• No response: 29.2%<br>• No serious adverse events	Single-arm study, short-term follow-up; no control group; recurrence not assessed
Cryotherapy	van Siegenbeek Heukelom et al. [62]	Retrospective, non-randomized study	HIV-positive MSM, n = 64	• Overall response (CR + PR) 60%<br>• Recurrence in 68% within 18 months<br>• Well tolerated with mostly mild adverse effects	Retrospective; small sample size; frequent recurrences; possible selection bias
Argon plasma coagulation	de Pokomandy et al. [52]	Prospective phase II, open-label pilot study	MSM living with HIV, n = 20	• HSIL Clearance at 24 months after repeated treatments: 65%<br>• Sustained HSIL-free status after 2-years: 35%	Very small pilot study, only 20 patients
Photodynamic therapy	van der Snoek et al. [63]	Prospective interventional study	HIV-positive MSM with AIN 3, n = 15; treatment sessions = 25	• Initial complete response: 28%<br>• Persistent complete response: 16%;<br>• Recurrence: 64% of initial responders after a mean follow-up of 8 months<br>• Significant treatment-related toxicity	Small single-center study; repeated treatment sessions, significant treatment-related toxicity
	Welbourn et al. [64]	Retrospective pilot study	Mixed AIN grades, n = 15 (10 available for outcome data)	• CR: 60% (6/10)<br>• PR: 30% (3/10);<br>• Recurrence: 50% (3/6) complete responders<br>• no major treatment-related complications	Very small retrospective study; heterogeneous population; only 10 patients evaluable for treatment response; repeated treatment sessions

MSM = Men who have sex with men, PLWH = People living with HIV

REFERENCES

[1] Solomon D, Davey D, Kurman R, Moriarty A, O'Connor D, Prey M et al. The 2001 Bethesda System: terminology for reporting results of cervical cytology. JAMA 2002;287(16):2114–9. https://doi.org/10.1001/jama.287.16.2114.

[2] Nayar R, Wilbur DC. The Pap test and Bethesda 2014. Cancer Cytopathol 2015;123(5):271–81. https://doi.org/10.1002/cncy.21521.

[3] Liu Y, McCluggage WG, Darragh TM, Zheng W, Roberts JM, Park KJ et al. Classifying Anal Intraepithelial Neoplasia 2 Based on LAST Recommendations. Am J Clin Pathol 2021;155(6):845–52. https://doi.org/10.1093/ajcp/aqaa188.

[4] Darragh TM, Colgan TJ, Thomas Cox J, Heller DS, Henry MR, Luff RD et al. The Lower Anogenital Squamous Terminology Standardization project for HPV-associated lesions: background and consensus recommendations from the College of American Pathologists and the American Society for Colposcopy and Cervical Pathology. Int J Gynecol Pathol 2013;32(1):76–115. https://doi.org/10.1097/PGP.0b013e31826916c7.

[5] Chromy D, Aigner F, Becker JC, Bickel M, Brunner A, Classen J et al. German-Austrian guideline on screening for anal dysplasia and anal carcinoma in people living with HIV. J Dtsch Dermatol Ges 2025. https://doi.org/10.1111/ddg.15719.

[6] van der Zee RP, Richel O, van Noesel CJM, Ciocănea-Teodorescu I, van Splunter AP, Braak TJ ter et al. Cancer Risk Stratification of Anal Intraepithelial Neoplasia in Human Immunodeficiency Virus-Positive Men by Validated Methylation Markers Associated With Progression to Cancer. Clin Infect Dis 2021;72(12):2154–63. https://doi.org/10.1093/cid/ciaa397.

[7] Silling S, Kreuter A, Hellmich M, Swoboda J, Pfister H, Wieland U. Human papillomavirus oncogene mRNA testing for the detection of anal dysplasia in HIV-positive men who have sex with men. J Clin Virol 2012;53(4):325–31. https://doi.org/10.1016/j.jcv.2011.12.029.

[8] Dupin C, Siproudhis L, Henno S, Minjolle S, Arvieux C, Tattevin P. Use of human papillomavirus genotyping and biomarkers for targeted screening of anal dysplasia in human immunodeficiency virus-infected patients. Dig Liver Dis 2015;47(5):423–8. https://doi.org/10.1016/j.dld.2015.01.150.

[9] Roberts JM, Poynten IM, Molano M, Machalek DA, Hillman RJ, Guzman P et al. Human Papillomavirus Genotypes in Anal High-Grade Squamous Intraepithelial Lesion (HSIL): Anal Intraepithelial Neoplasia Grades 2 (AIN2) and 3 (AIN3) Are Different. Cancer Epidemiol Biomarkers Prev 2020;29(10):2078–83. https://doi.org/10.1158/1055-9965.EPI-20-0664.

[10] Lyons KM, Butler SL. Anal Intraepithelial Neoplasia from a Pathologists Point of View. Clin Colon Rectal Surg 2018;31(6):328–35. https://doi.org/10.1055/s-0038-1668102.

[11] Thomas M, Pim D, Banks L. The role of the E6-p53 interaction in the molecular pathogenesis of HPV. Oncogene 1999;18(53):7690–700. https://doi.org/10.1038/sj.onc.1202953.

[12] Yeo-Teh NSL, Ito Y, Jha S. High-Risk Human Papillomaviral Oncogenes E6 and E7 Target Key Cellular Pathways to Achieve Oncogenesis. Int J Mol Sci 2018;19(6). https://doi.org/10.3390/ijms19061706.

[13] Liu Y, Blakely M, Sigel K, Thin TH, Hui P, Donovan M et al. Biomarker P16 predicts progression risk of anal low-grade squamous intraepithelial lesions. AIDS 2018;32(16):2309–16. https://doi.org/10.1097/QAD.0000000000001957.

[14] Doorbar J, Quint W, Banks L, Bravo IG, Stoler M, Broker TR et al. The biology and life-cycle of human papillomaviruses. Vaccine 2012;30 Suppl 5:F55-70. https://doi.org/10.1016/j.vaccine.2012.06.083.

[15] Leclerc E, Jacomet C, Siproudhis L, Abramowitz L, Pereira B, Buisson A. Impact of screening programme to prevent anal cancer in high-risk patients with HIV. HIV Med 2024;25(4):454–61. https://doi.org/10.1111/hiv.13594.
[16] Lee GC, Kunitake H, Milch H, Savitt LR, Stafford CE, Bordeianou LG et al. What Is the Risk of Anal Carcinoma in Patients With Anal Intraepithelial Neoplasia III? Dis Colon Rectum 2018;61(12):1350–6. https://doi.org/10.1097/DCR.0000000000001219.
[17] Faber MT, Frederiksen K, Palefsky JM, Kjaer SK. A nationwide longitudinal study on risk factors for progression of anal intraepithelial neoplasia grade 3 to anal cancer. Int J Cancer 2022;151(8):1240–7. https://doi.org/10.1002/ijc.34143.
[18] Sendagorta Cudós E, Sotomayor C, Masia Canuto M, Cabello A, Curran A, Ocampo A et al. Incidence of Anal Cancer and Related Risk Factors in HIV-Infected Patients Enrolled in the National Prospective Spanish Cohort CoRIS. Dis Colon Rectum 2023;66(12):e1186-e1194. https://doi.org/10.1097/DCR.0000000000002940.
[19] Haas CB, Engels EA, Palefsky JM, Clarke MA, Kreimer AR, Luo Q et al. Severe anal intraepithelial neoplasia trends and subsequent invasive anal cancer in the United States. J Natl Cancer Inst 2024;116(1):97–104. https://doi.org/10.1093/jnci/djad176.
[20] Palefsky JM, Lee JY, Jay N, Goldstone SE, Darragh TM, Dunlevy HA et al. Treatment of Anal High-Grade Squamous Intraepithelial Lesions to Prevent Anal Cancer. N Engl J Med 2022;386(24):2273–82. https://doi.org/10.1056/NEJMoa2201048.
[21] Scholefield JH, Castle MT, Watson NFS. Malignant transformation of high-grade anal intraepithelial neoplasia. Br J Surg 2005;92(9):1133–6. https://doi.org/10.1002/bjs.4994.
[22] Watson AJM, Smith BB, Whitehead MR, Sykes PH, Frizelle FA. Malignant progression of anal intra-epithelial neoplasia. ANZ J Surg 2006;76(8):715–7. https://doi.org/10.1111/j.1445-2197.2006.03837.x.
[23] Albuquerque A, Fontes F. Recent Guidelines on Anal Cancer Screening: A Systematic Review. J Low Genit Tract Dis 2025. https://doi.org/10.1097/LGT.0000000000000878.
[24] Espirito Santo I, Kefleyesus A, Chilou C, Faes S, Clerc D, Hübner M et al. Anal Cancer Screening: 10-Year Experience of a Specialized Outpatient Clinic. Cancers 2025;17(2). https://doi.org/10.3390/cancers17020193.
[25] Dias Gonçalves Lima F, Viset JD, Leeflang MMG, Limpens J, Prins JM, Vries HJC de. The Accuracy of Anal Swab-Based Tests to Detect High-Grade Anal Intraepithelial Neoplasia in HIV-Infected Patients: A Systematic Review and Meta-analysis. Open Forum Infect Dis 2019;6(5):ofz191. https://doi.org/10.1093/ofid/ofz191.
[26] Gaisa MM, Sigel KM, Deshmukh AA, Lenskaya V, Chan CA, Silvera R et al. Comparing Anal Cancer Screening Algorithms Using Cytology and Human Papillomavirus DNA Testing in 3 High-Risk Populations. J Infect Dis 2021;224(5):881–8. https://doi.org/10.1093/infdis/jiaa801.
[27] Stewart DB, Gaertner WB, Glasgow SC, Herzig DO, Feingold D, Steele SR. The American Society of Colon and Rectal Surgeons Clinical Practice Guidelines for Anal Squamous Cell Cancers (Revised 2018). Dis Colon Rectum 2018;61(7):755–74. https://doi.org/10.1097/DCR.0000000000001114.
[28] Binda GA, Gagliardi G, Dal Conte I, Verra M, Cassoni P, Cavazzoni E et al. Practice parameters for the diagnosis and treatment of anal intraepithelial neoplasia (AIN) on behalf of the Italian Society of Colorectal Surgery (SICCR). Tech Coloproctol 2019;23(6):513–28. https://doi.org/10.1007/s10151-019-02019-5.
[29] Berry JM, Palefsky JM, Jay N, Cheng S-C, Darragh TM, Chin-Hong PV. Performance characteristics of anal cytology and human papillomavirus testing in patients with high-resolution anoscopy-guided biopsy of high-grade anal intraepithelial neoplasia. Dis Colon Rectum 2009;52(2):239–47. https://doi.org/10.1007/DCR.0b013e31819793d9.

[30] Dalla Pria A, Alfa-Wali M, Fox P, Holmes P, Weir J, Francis N et al. High-resolution anoscopy screening of HIV-positive MSM: longitudinal results from a pilot study. AIDS 2014;28(6):861–7. https://doi.org/10.1097/QAD.0000000000000160.
[31] Chiao EY, Giordano TP, Palefsky JM, Tyring S, El Serag H. Screening HIV-infected individuals for anal cancer precursor lesions: a systematic review. Clin Infect Dis 2006;43(2):223–33. https://doi.org/10.1086/505219.
[32] Silvera R, Martinson T, Gaisa MM, Liu Y, Deshmukh AA, Sigel K. The other side of screening: predictors of treatment and follow-up for anal precancers in a large health system. AIDS 2021;35(13):2157–62. https://doi.org/10.1097/QAD.0000000000002948.
[33] Stier EA, Clarke MA, Deshmukh AA, Wentzensen N, Liu Y, Poynten IM et al. International Anal Neoplasia Society's consensus guidelines for anal cancer screening. Int J Cancer 2024;154(10):1694–702. https://doi.org/10.1002/ijc.34850.
[34] Wei F, Alberts CJ, Albuquerque A, Clifford GM. Impact of Human Papillomavirus Vaccine Against Anal Human Papillomavirus Infection, Anal Intraepithelial Neoplasia, and Recurrence of Anal Intraepithelial Neoplasia: A Systematic Review and Meta-analysis. J Infect Dis 2023;228(11):1496–504. https://doi.org/10.1093/infdis/jiad183.
[35] Palefsky JM, Giuliano AR, Goldstone S, Moreira ED, Aranda C, Jessen H et al. HPV vaccine against anal HPV infection and anal intraepithelial neoplasia. N Engl J Med 2011;365(17):1576–85. https://doi.org/10.1056/NEJMoa1010971.
[36] Goldstone SE, Giuliano AR, Palefsky JM, Lazcano-Ponce E, Penny ME, Cabello RE et al. Efficacy, immunogenicity, and safety of a quadrivalent HPV vaccine in men: results of an open-label, long-term extension of a randomised, placebo-controlled, phase 3 trial. Lancet Infect Dis 2022;22(3):413–25. https://doi.org/10.1016/S1473-3099(21)00327-3.
[37] Muñoz N, Manalastas R, Pitisuttithum P, Tresukosol D, Monsonego J, Ault K et al. Safety, immunogenicity, and efficacy of quadrivalent human papillomavirus (types 6, 11, 16, 18) recombinant vaccine in women aged 24-45 years: a randomised, double-blind trial. Lancet 2009;373(9679):1949–57. https://doi.org/10.1016/S0140-6736(09)60691-7.
[38] Patel C, Brotherton JM, Pillsbury A, Jayasinghe S, Donovan B, Macartney K et al. The impact of 10 years of human papillomavirus (HPV) vaccination in Australia: what additional disease burden will a nonavalent vaccine prevent? Euro Surveill 2018;23(41). https://doi.org/10.2807/1560-7917.ES.2018.23.41.1700737.
[39] Baandrup L, Maltesen T, Dehlendorff C, Kjaer SK. Human papillomavirus vaccination and anal high-grade precancerous lesions and cancer-a real-world effectiveness study. J Natl Cancer Inst 2024;116(2):283–7. https://doi.org/10.1093/jnci/djad189.
[40] Wilkin TJ, Chen H, Cespedes MS, Leon-Cruz JT, Godfrey C, Chiao EY et al. A Randomized, Placebo-Controlled Trial of the Quadrivalent Human Papillomavirus Vaccine in Human Immunodeficiency Virus-Infected Adults Aged 27 Years or Older: AIDS Clinical Trials Group Protocol A5298. Clin Infect Dis 2018;67(9):1339–46. https://doi.org/10.1093/cid/ciy274.
[41] Palefsky JM, Berry JM, Jay N, Krogstad M, Da Costa M, Darragh TM et al. A trial of SGN-00101 (HspE7) to treat high-grade anal intraepithelial neoplasia in HIV-positive individuals. AIDS 2006;20(8):1151–5. https://doi.org/10.1097/01.aids.0000226955.02719.26.
[42] Klencke B, Matijevic M, Urban RG, Lathey JL, Hedley ML, Berry M et al. Encapsulated plasmid DNA treatment for human papillomavirus 16-associated anal dysplasia: a Phase I study of ZYC101. Clin Cancer Res 2002;8(5):1028–37.
[43] Gosens KCM, van der Zee RP, van Heukelom MLS, Jongen VW, Cairo I, van Eeden A et al. HPV vaccination to prevent recurrence of anal intraepithelial neoplasia in HIV+ MSM. AIDS 2021;35(11):1753–64. https://doi.org/10.1097/QAD.0000000000002928.

[44] Hidalgo-Tenorio C, Pasquau J, Omar-Mohamed M, Sampedro A, López-Ruz MA, López Hidalgo J et al. Effectiveness of the Quadrivalent HPV Vaccine in Preventing Anal ≥ HSILs in a Spanish Population of HIV+ MSM Aged 26 Years. Viruses 2021;13(2). https://doi.org/10.3390/v13020144.
[45] Kechagias KS, Kalliala I, Bowden SJ, Athanasiou A, Paraskevaidi M, Paraskevaidis E et al. Role of human papillomavirus (HPV) vaccination on HPV infection and recurrence of HPV related disease after local surgical treatment: systematic review and meta-analysis. BMJ 2022;378:e070135. https://doi.org/10.1136/bmj-2022-070135.
[46] Gosens KCM, van der Burg SH, Welters MJP, Boekestijn S, Loof NM, Quint WGV et al. Therapeutic Vaccination against Human Papillomavirus Type 16 for the Treatment of High-Grade Anal Intraepithelial Neoplasia in HIV+ Men. Clin Cancer Res 2023;29(20):4109–17. https://doi.org/10.1158/1078-0432.CCR-22-3361.
[47] Burgos J, Curran A, Garcia J, Landolfi S, Hernández-Losa J, Suanzes P et al. Effectiveness of trichloroacetic acid versus electrocautery for the treatment of anal high-grade squamous intraepithelial lesions in persons with HIV. Infect Dis (Lond) 2024;56(4):299–307. https://doi.org/10.1080/23744235.2024.2303021.
[48] Goldstone SE, Terlizzi JP, Levine RA, Moshier E, Pereira Vera B. A Prospective, Dual-Center Trial of Circumferential Radiofrequency Ablation of Anal High-Grade Squamous Intraepithelial Lesions Demonstrate Improved Long-term Efficacy Over Historical Controls of Targeted Ablation. Dis Colon Rectum 2023;66(6):764–73. https://doi.org/10.1097/DCR.0000000000002365.
[49] Fuertes I, Chivite I, Cranston RD, Sánchez E, Cordón E, Rodríguez-Carunchio L et al. Short-term effectiveness and tolerability of carbon dioxide laser for anal high-grade squamous intraepithelial lesions in individuals living with HIV. Int J STD AIDS 2022;33(7):709–17. https://doi.org/10.1177/09564624221100069.
[50] Hidalgo-Tenorio C, García-Martínez CM, Pasquau J, Omar-Mohamed-Balgahata M, López-Ruz M, López-Hidalgo J et al. Risk factors for ≥high-grade anal intraepithelial lesions in MSM living with HIV and the response to topical and surgical treatments. PLoS One 2021;16(2):e0245870. https://doi.org/10.1371/journal.pone.0245870.
[51] Fuertes I, Bastida C, Lopez-Cabezas C, Rodríguez-Carunchio L, Ordi J, Mallolas J et al. The effectiveness and tolerability of imiquimod suppositories to treat extensive intra-anal high-grade squamous intraepithelial lesions/warts in HIV-infected individuals. Int J STD AIDS 2019;30(12):1194–200. https://doi.org/10.1177/0956462419864506.
[52] Pokomandy A de, Rouleau D, Lalonde R, Beauvais C, Castro C de, Coutlée F. Argon plasma coagulation treatment of anal high-grade squamous intraepithelial lesions in men who have sex with men living with HIV: results of a 2-year prospective pilot study. HIV Med 2018;19(2):81–9. https://doi.org/10.1111/hiv.12544.
[53] Goldstone RN, Hasan SR, Drury S, Darragh TM, van Zante A, Goldstone SE. A trial of radiofrequency ablation for anal intraepithelial neoplasia. Int J Colorectal Dis 2017;32(3):357–65. https://doi.org/10.1007/s00384-016-2679-2.
[54] Burgos J, Curran A, Landolfi S, Navarro J, Tallada N, Guelar A et al. The effectiveness of electrocautery ablation for the treatment of high-grade anal intraepithelial neoplasia in HIV-infected men who have sex with men. HIV Med 2016;17(7):524–31. https://doi.org/10.1111/hiv.12352.
[55] Stier EA, Goldstone SE, Einstein MH, Jay N, Berry JM, Wilkin T et al. Safety and efficacy of topical cidofovir to treat high-grade perianal and vulvar intraepithelial neoplasia in HIV-positive men and women. AIDS 2013;27(4):545–51. https://doi.org/10.1097/QAD.0b013e32835a9b16.

[56] Weis SE, Vecino I, Pogoda JM, Susa JS. Treatment of high-grade anal intraepithelial neoplasia with infrared coagulation in a primary care population of HIV-infected men and women. Dis Colon Rectum 2012;55(12):1236–43. https://doi.org/10.1097/DCR.0b013e31826d5cb5.

[57] Richel O, Wieland U, Vries HJC de, Brockmeyer NH, van Noesel C, Potthoff A et al. Topical 5-fluorouracil treatment of anal intraepithelial neoplasia in human immunodeficiency virus-positive men. Br J Dermatol 2010;163(6):1301–7. https://doi.org/10.1111/j.1365-2133.2010.09982.x.

[58] Wieland U, Brockmeyer NH, Weissenborn SJ, Hochdorfer B, Stücker M, Swoboda J et al. Imiquimod treatment of anal intraepithelial neoplasia in HIV-positive men. Arch Dermatol 2006;142(11):1438–44. https://doi.org/10.1001/archderm.142.11.1438.

[59] Chang GJ, Berry JM, Jay N, Palefsky JM, Welton ML. Surgical treatment of high-grade anal squamous intraepithelial lesions: a prospective study. Dis Colon Rectum 2002;45(4):453–8. https://doi.org/10.1007/s10350-004-6219-8.

[60] Howard A, Joe T, Mitchell T, Truong PT. The efficacy of radiotherapy in perianal intraepithelial neoplasia grade 3. World Cancer Research Journal. 2024. 2021;8:e1963.

[61] Ortholan C, Ramaioli A, Peiffert D, Lusinchi A, Romestaing P, Chauveinc L et al. Anal canal carcinoma: early-stage tumors < or =10 mm (T1 or Tis): therapeutic options and original pattern of local failure after radiotherapy. Int J Radiat Oncol Biol Phys 2005;62(2):479–85. https://doi.org/10.1016/j.ijrobp.2004.09.060.

[62] van Siegenbeek Heukelom ML, Gosens KCM, Prins JM, Vries HJC de. Cryotherapy for Intra- and Perianal High-Grade Squamous Intraepithelial Lesions in HIV-Positive Men who have Sex with Men. Am J Clin Dermatol 2018;19(1):127–32. https://doi.org/10.1007/s40257-017-0311-z.

[63] van der Snoek EM, Hollander JC den, Aans JB, Sterenborg HJCM, van der Ende ME, Robinson DJ. Photodynamic therapy with systemic meta-tetrahydroxyphenylchlorin in the treatment of anal intraepithelial neoplasia, grade 3. Lasers Surg Med 2012;44(8):637–44. https://doi.org/10.1002/lsm.22062.

[64] Welbourn H, Duthie G, Powell J, Moghissi K. Can photodynamic therapy be the preferred treatment option for anal intraepithelial neoplasia? Initial results of a pilot study. Photodiagnosis Photodyn Ther 2014;11(1):20–1. https://doi.org/10.1016/j.pdpdt.2013.11.004.
